# Inhibitory Effect of CAPE and Kaempferol in Colon Cancer Cell Lines—Possible Implications in New Therapeutic Strategies

**DOI:** 10.3390/ijms20051199

**Published:** 2019-03-09

**Authors:** Liviuta Budisan, Diana Gulei, Ancuta Jurj, Cornelia Braicu, Oana Zanoaga, Roxana Cojocneanu, Laura Pop, Lajos Raduly, Alexandru Barbat, Alin Moldovan, Cristian Moldovan, Adrian Bogdan Tigu, Calin Ionescu, Atanas G. Atanasov, Alexandru Irimie, Ioana Berindan-Neagoe

**Affiliations:** 1 Research Center for Functional Genomics, Biomedicine and Translational Medicine, “Iuliu Hatieganu” University of Medicine and Pharmacy, 23 Marinescu Street, 400337 Cluj-Napoca, Romania; liviutabudisan@gmail.com (L.B.); ancajurj15@gmail.com (A.J.); braicucornelia@yahoo.com (C.B.); zanoaga.oana@gmail.com (O.Z.); cojocneanur@gmail.com (R.C.); laura.ancuta.pop@gmail.com (L.P.); raduly.lajos78@gmail.com (L.R.); barbatalexandruteodor@gmail.com (A.B.); 2 MEDFUTURE - Research Center for Advanced Medicine, “Iuliu-Hatieganu” University of Medicine and Pharmacy, 23 Marinescu Street, 400337 Cluj-Napoca, Romania; diana.c.gulei@gmail.com (D.G.); alin.moldovan92@yahoo.ro (A.M.); moldovan.cristian1994@gmail.com (C.M.); adrianbogdantigu@gmail.com (A.B.T.); 3 5th Surgical Department, Municipal Hospital, 400139 Cluj-Napoca, Romania; 4 “Iuliu Hatieganu” University of Medicine and Pharmacy, 400000 Cluj-Napoca, Romania; 5 Institute of Genetics and Animal Breeding of the Polish Academy of Sciences, 05-552 Jastrzebiec, Poland; atanas.atanasov@univie.ac.at; 6 Department of Pharmacognosy, University of Vienna, Althanstrasse 14, A-1090 Vienna, Austria; 7 11th Department of Oncological Surgery and Gynecological Oncology, “Iuliu Hatieganu” University of Medicine and Pharmacy, 400015 Cluj-Napoca, Romania; a.irimie@umfcluj.ro; 8 Department of Surgery, The Oncology Institute “Prof. Dr. Ion Chiricuta”, 400015 Cluj-Napoca, Romania; 9 Department of Functional Genomics and Experimental Pathology, The Oncology Institute “Prof. Dr. Ion Chiricuta”, 34-36 Republicii Street, 400015 Cluj-Napoca, Romania

**Keywords:** natural compounds, polyphenols, colon cancer, CAPE, Kaempferol, Morin

## Abstract

Background: Phytochemicals are natural compounds synthesized as secondary metabolites in plants and represent an important source of molecules with therapeutic applications. Attention is accorded to their potential in anti-cancer therapies as single agents or adjuvant treatment. Herby, we evaluated the in vitro effects of a panel of natural compounds with focus on caffeic acid phenethyl ester (CAPE) and Kaempferol for the treatment of human colon cancer. Methods: We exposed two human colon cancer cell lines, RKO and HCT-116, followed by functional examination of cell viability, cell proliferation and invasion, cell cycle, apoptosis, and autophagy. Modifications in gene expression were investigated through microarray and detection of existing mutations and finding of new ones was done with the help of Next Generation Sequencing (NGS). Results: Both CAPE and Kaempferol inhibit cell proliferation, motility and invasion, and stimulate apoptosis and autophagy, concomitant with modifications in coding and noncoding genes’ expression. Moreover, there are pathogenic mutations that are no longer found upon treatment with CAPE and Kaempferol. Conclusions: Our findings indicate that CAPE and Kaempferol have the ability to negatively influence the development and advancement of colon cancer in vitro by specifically altering the cells at the molecular level; this activity can be exploited in possible adjuvant therapies once the optimal dose concentration with minimal side effects but with cancer inhibitory activity is set in vivo.

## 1. Introduction

According to Globocan 2018 data, colon cancer is ranking as the third most common site of cancer and the second one in terms of mortality [1]. These concerning statistics are mainly caused by late diagnosis, but also by unresponsive phenotypes to the current standard of care; moreover, these two parameters are resulting in a worse outcome when combined [2]. Therefore, introduction of new efficient treatment options or addition of adjuvant molecules to the current care is one of the main strategies that have to be investigated in this pathology, together with implementation of early diagnosis methods. 

Phytochemicals are secondary metabolites naturally found in plants with roles in restoration of damaged cells, but also in determination of colour, aroma, and taste of plants/fruits. These products are classified based on their biosynthesis starting point: phenolic compounds, carotenoids, products with nitrogen, alkaloids, and organosulfur compounds [3,4,5,6,7]. Despite being one of the oldest treatment schemes for a wide range of pathologies, their roles are reinvented today with the help of last generation instruments able to deliver molecular data regarding their therapeutic effects [8,9]. Initially associated with antioxidant properties and ability to prevent the formation of free radicals, current studies reveal a more complex protective action at cellular and molecular levels, with important application in disease prevention or treatment. These evidences are also supported by epidemiological data [9,10]. A steady advantage of these compounds consists of their safety profile, limited side effects, bioavailability, and cost effectiveness. Even if the impact of polyphenols was demonstrated in the beginning of traditional medicine, nowadays they are associated with modifications in coding and noncoding gene expression or epigenetic events [7,9]; these novel data are backed up by the latest progresses in the “omics” approaches [11]. Moreover, an emerging number of studies are focused on delivery options that will allow the concentration of the active compound at the tumour level, concomitant with increased therapeutic efficiency, decreased dose dispersion and limitation of side effects [12,13]. Advancing in the world of natural compounds, there are studies focused on delivery of phytochemicals, specifically curcumin, via plant exosomes to colon cancer tissue [14]. 

Caffeic acid phenethyl ester (CAPE) is one of the most important bioactive agents found in high concentration in propolis. CAPE has multiple biologic properties, including antiviral, antibacterial, antioxidant, and anti-inflammatory activity [7,15,16,17]. However, a limited number of studies has approached the effects of CAPE in colon cancer [18]. Kaempferol is a natural flavonol with antioxidant properties found in a variety of plants and plant-derived foods. Multiple investigations have confirmed the cancer inhibitory role of this compound [7,19,20,21,22]. 

In the present study, we used CAPE and Kampferol to investigate their inhibitory role on colon cancer cell lines through functional evaluation of the treated cells and interrogation of DNA mutations and gene expression (coding and noncoding). 

## 2. Results

### 2.1. Evaluation of Colon Cancer Cell Viability after Treatment with Natural Compounds in Different Doses 

**Figure 1:**
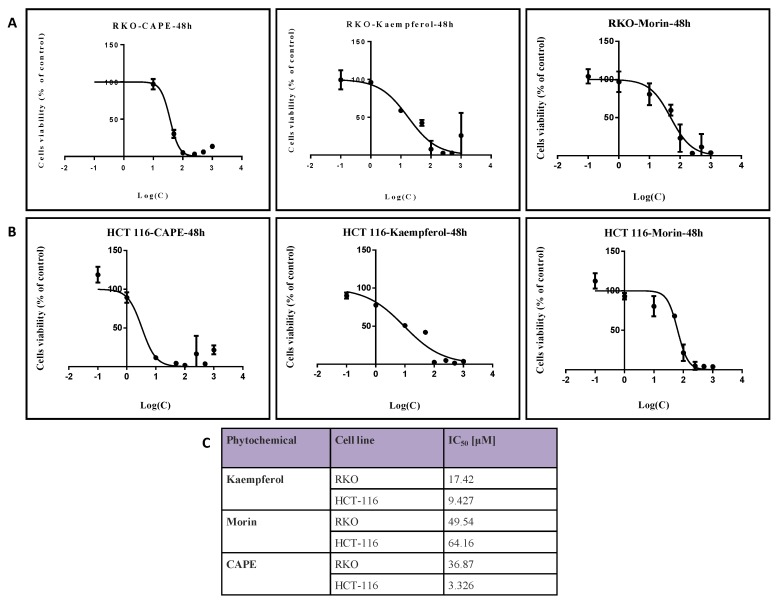
Cell viability. Measurement of colon cell viability after treatment for 48 h with CAPE, Kaempherol and Morin in (**A**) RKO and (**B**) HCT-116 cell lines. (**C**) Quantification of IC_50_ corresponding dose for each of the compound in the two colon cancer cell lines. Dose concentrations (µM) were calculated in logarithmic format for graphical representation. C—concentration.

In order to assess the compounds with potential therapeutic value in colon cancer we tested the efficiency of CAPE, Kaempferol, Morin, EGCG, Daidzein, and Genistein in different concentrations on RKO, HCT-116, HT-29, and DLD-1 colon cancer cell lines in terms of inhibition of cell viability in a dose-dependent manner. The compounds showed different inhibitory levels in dependency with their structure, cell line, and dose (Supplementary Figure S1). After evaluation of the entire panel of tested compounds, we observed that CAPE, Kaempferol, and Morin hold the lowest IC_50_ values with cancer inhibitory effects in RKO and HCT-116 cell lines (Figure 1). Moreover, the most efficient compound in the impairment of colon cancer cell viability in balance with the effect/dose ratio was CAPE in HCT-116, followed by Kaempferol in the same cell line. Taken as general effects in colon cancer (meaning the inhibitory role upon both of the cell lines was in concordance with used doses), Kaempferol showed the most potent activity. It was decided to continue the functional evaluation on RKO and HCT-116 cell lines, with these two lines having better treatment responses. 

### 2.2. Cell Cycle Evaluation for RKO and HCT-116 Treated with Cape, Morin, and Kaempherol

**Figure 2:**
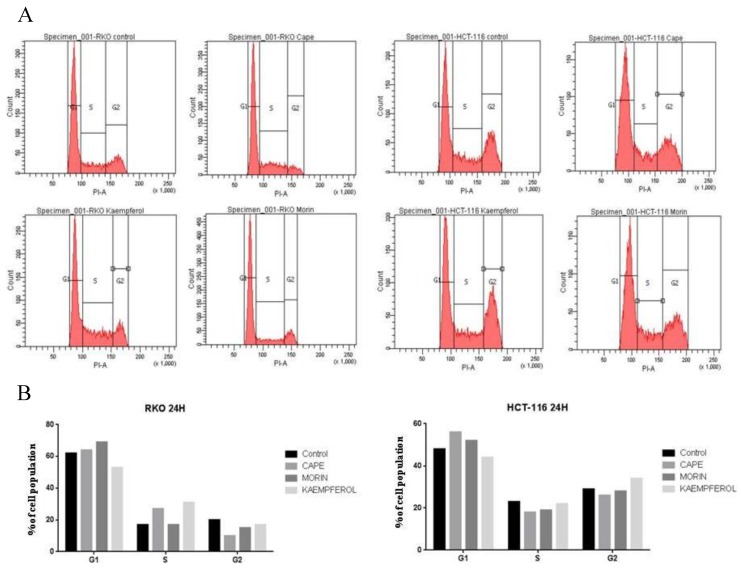
Cell cycle evaluation after treatment with CAPE, Kaempherol, and Morin. (**A**) The weight of G1, S, G2 cell cycle phases in the RKO population without treatment (Control), treated with 36.87 µM of CAPE, 17.42 µM of Kaempherol, and 49.54 µM of Morin for 24 h. (**B**) The weight of G1, S, G2 cell cycle phases in the HCT-116 population without treatment (Control), treated with 3.326 µM of CAPE, 9.427 µM of Kaempherol, and 64.16 µM of Morin for 24 h. % of cell population—percent of cells within the same sample corresponding to the specific cell cycle phase (G1, S, G2).

The flow cytometry results for cell cycle evaluation in the RKO cell line were obtained 24 h after treatment with each compound—Cape, Morin, and Kaempherol—in doses corresponding to their specific IC_50_ value (Figure 2A). As observed, the majority of the cell population is in G1 phase, having a low division rate; among all, Morin has the most pronounced effects in increasing the number of cells in G1 phase compared to control counterparts. For the same compound, there are minimal effects for the S phase accumulation in relationship with control and a decrease in the number of cells situated in the G2 phase. For the HCT-116 cell line treated with the same compounds for 24 h (Figure 2B), the most pronounced effects were observed in the case of CAPE compared to control, where the number of cells in G1 phase increased; these results correlate with an effect of stopping the cell cycle. This ability is especially important in the case of CAPE considering the minimal dose used for the cell treatment, 3.326 µM, showing the potential of this molecule in impairing in vitro cancer spreading. Inversed results are obtained for Kaempherol, with a smaller proportion of cells in G1 phase and an increased number in G2 phase. Morin also showed potential in modifying the cell cycle in HCT-116 cells. 

### 2.3. Assessment of Cell Invasion for RKO and HCT-116 Cells Treated with Cape, Morin, and Kaempherol

**Figure 3:**
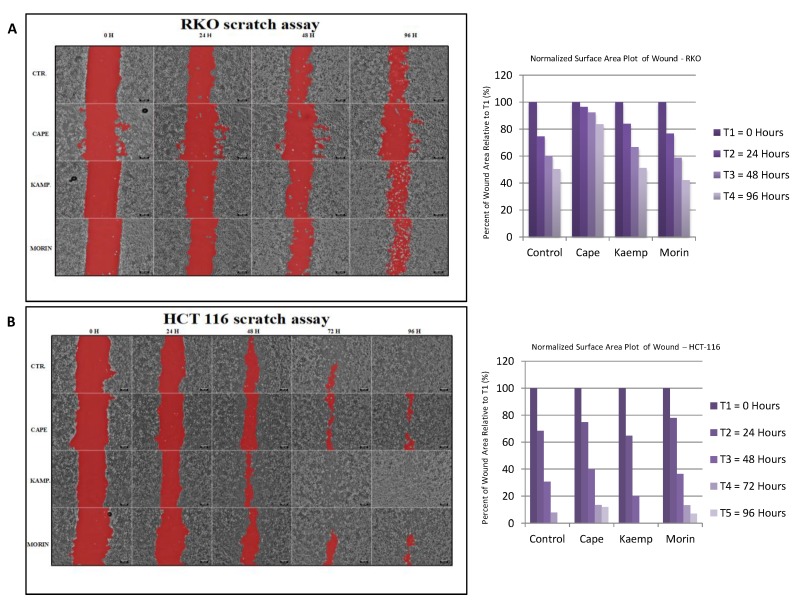
Invasion evaluation after treatment with CAPE, Kaempherol, and Morin. Wound healing assay performed for (**A**) RKO cells without treatment (Control, CTR.) and treated with 36.87 µM of CAPE, 17.42 µM of Kaempherol, and 49.54 µM of Morin for 48 h, together with graphical representation of the scratch measurement at 0, 24, 48, and 96 h. Wound healing assay performed for (**B**) HCT-116 cells without treatment (Control, CTR.) and treated with 3.326 µM of CAPE, 9.427 µM of Kaempherol and 64.16 µM of Morin for 48 h, together with graphical representation of the scratch measurement at 0, 24, 48, 72, and 96 h. Pictures were taken with Olympus IX71 microscope using cellSens software; scale bar was set at 200 µM.

To evaluate the effect of CAPE, Morin, and Kaempherol in limiting the invasion of colon cancer cells in vitro, we performed a wound assay for control and treated cells and followed the gap closure for 96 h. Concomitant with cell cycle analysis, CAPE managed to slow down the cell invasion in both cell lines (Figure 3A,B), where a larger gap was still present at 96 h in comparison with the control group. The other two compounds, Kaempherol and Morin, were not associated with marked differences in terms of cell invasiveness.

### 2.4. Apoptosis and Authophagy upon Treatment with Cape and Kaempherol 

**Figure 4:**
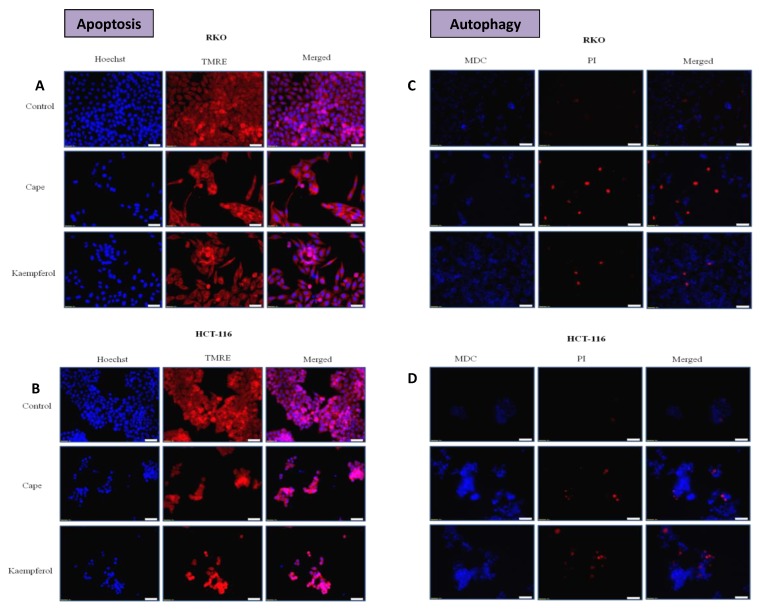
Apoptosis and autophagy in colon cancer cells after treatment with CAPE and Kaempherol. Apoptosis (TMRE/Hoechst) was evaluated in (**A**) RKO cells without treatment (Control) and treated with 36.87 µM of CAPE and 17.42 µM of Kaempherol for 48 h and in (**B**) HCT-116 cells without treatment (Control) and treated with 3.326 µM of CAPE and 9.427 µM of Kaempherol for 48 h. Autophagy (PI/MDC) was evaluated in (**C**) RKO cells without treatment (Control) and treated with 36.87 µM of CAPE and 17.42 µM of Kaempherol for 48 h and in (**D**) HCT-116 cells without treatment (Control) and treated with 3.326 µM of CAPE and 9.427 µM of Kaempherol for 48 h. Pictures were taken with Olympus IX71 microscope using cellSens software; pictures were taken at a resolution of 20× (scale bar).

Following the investigation of cell cycle and invasion, the capacity of CAPE and Kaempherol to induce apoptosis or autophagy in RKO and HCT-116 colon cancer cell lines was assessed. Cells were treated and investigated in parallel with the control counterparts. In the case of apoptosis evaluation (Figure 4A for RKO and Figure 4B for HCT-116), Hoechst marking was used for nucleus, TMRE, and fluorochrome superposition. Decrease in TMRE fluorescence was considered a sign of apoptosis. For both cell lines, a decline in the number of marked nuclei was evident, especially for the case of HCT-116 cells treated with Kaempherol. 

For autophagy evaluation (Figure 4C for RKO and Figure 4D for HCT-116), monodansylcadaverine (MDC), an autophagy vacuole marker, was used, showing increased authophagy for cells treated with natural compounds in comparison with control ones. Despite the fact that autophagy is generally considered a survival mechanism, there is increasing evidence that degradation of essential cellular components during this signalling cascade can provoke the onset of cell death [23]. In our case, the coexistence of both autophagy and apoptosis, together with the previously shown decrease in cellular viability, could stand as an indicator that autophagy can function as a pre-existing state before cell death. However, additional research is needed for this particular field. 

**Figure 5:**
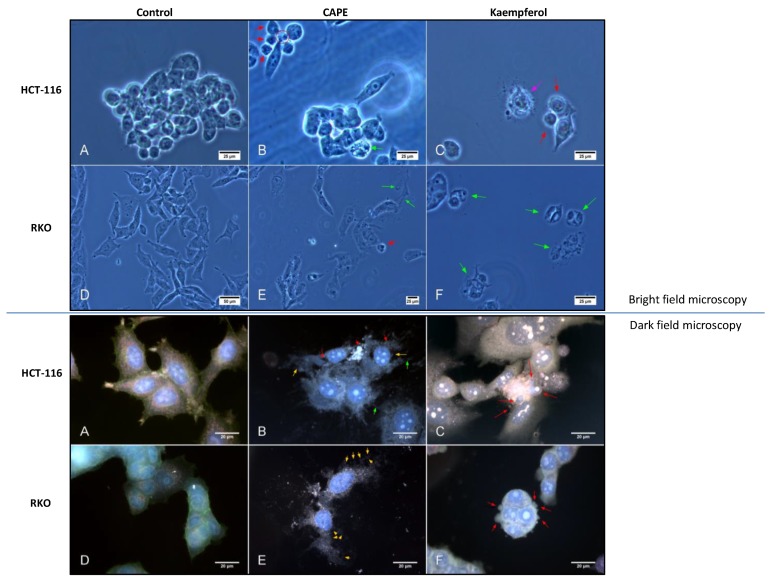
Bright and dark-field microscopy in colon cancer cells after treatment with CAPE and Kaempherol. HCT-116 cells (Bright field **A**,**B**,**C**; Dark field **A**,**B**,**C**) without treatment (Control) and treated with 3.326 µM of CAPE and 9.427 µM of Kaempherol for 48 h; RKO cells (Bright field **D**,**E**,**F**; Dark field **D**,**E**,**F**) without treatment (Control) and treated with 36.87 µM of CAPE and 17.42 µM of Kaempherol for 48 h. Bright-field microscopy: red arrows—apoptosis associated morphology, green arrows—necrosis associated morphology; pink arrow—membrane blebbing; red circle—apoptotic bodies; Dark-field microscopy: yellow arrows—irregular membrane, red arrows—apoptosis associated morphology; green arrows—possible apoptopodia or tunnelling nanotubes.

In order to investigate the induction of apoptosis in depth, bright and darkfield microscopy for HCT-116 and RKO cells treated with natural compounds (CAPE and Kaempferol) was performed (Figure 5). For phase contrast microscopy, red arrows point toward apoptotic cells, whereas green ones point toward cells with necrotic morphologies. In dark-field images, yellow arrows point toward areas of the membrane that are highly irregular or interrupted; red arrows indicate the presence of apoptotic bodies. The two green arrows point toward cellular projections that resemble apoptopodia or tunnelling nanotubes, but the degree of cellular degradation prevents a proper assessment. 

**Figure 6:**
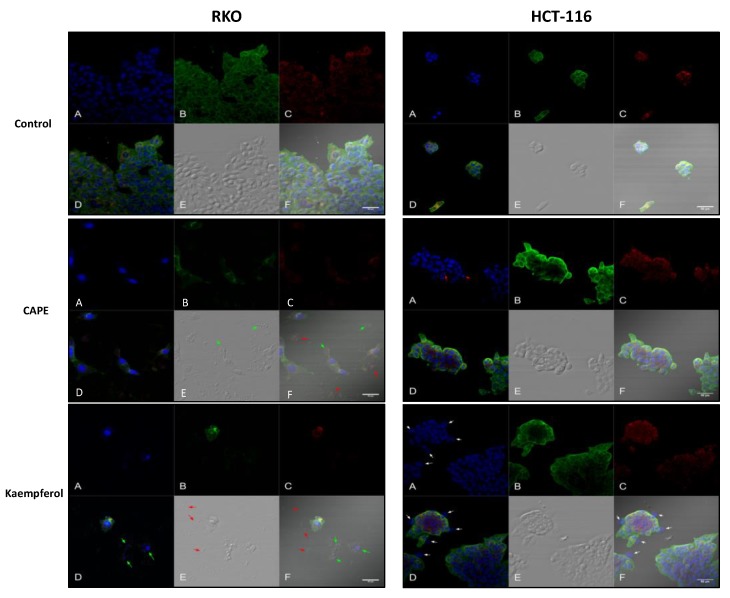
Confocal fluorescence microscopy for RKO and HCT 116 treated with Cape and Kaempherol. RKO cells without treatment (Control) and treated with 36.87 µM of CAPE and 17.42 µM of Kaempherol for 48 h; HCT-116 cells without treatment (Control) and treated with 3.326 µM of CAPE and 9.427 µM of Kaempherol for 48 h; (**A**) DAPI for nucleus, (**B**) FITC for cytoskeleton (F-Actine), (**C**) CytoPainter MitoRed for mitochondria, (**D**) Merged image, (**E**) NOMARSKI marked, (**F**) Merged image. Horizontal band pass filtered 1% tolerance, 2-pixel lower threshold on Nomarski. The contrast is modified for each colour of the histogram, intensity calibration at the upper limit of saturation on the detector. Pixel size 0.31 µm. RKO treated with CAPE: green arrows—cellular debris with mitochondria; red arrows—nucleus remnants with spherical aspect (necrosis sign); RKO treated with Kaempherol: green arrows—nucleus without cytoplasm; red arrows—cellular debris with cytoskeleton remnants (necrosis sign); HCT-116 treated with CAPE: red arrows—fragmented nuclei; HCT treated with Kaempherol: white arrows: enlarged nuclei.

Confocal fluorescence microscopy imaging showed induction of apoptosis for CAPE and Kaempferol treatment on both cell lines, confirming the results from bright and dark field microscopy (Figure 6). 

### 2.5. Microarray Analysis for Coding and Noncoding Genes’ Expression in RKO and HCT-116 Cell Line Treated with CAPE and Kaempherol

**Figure 7:**
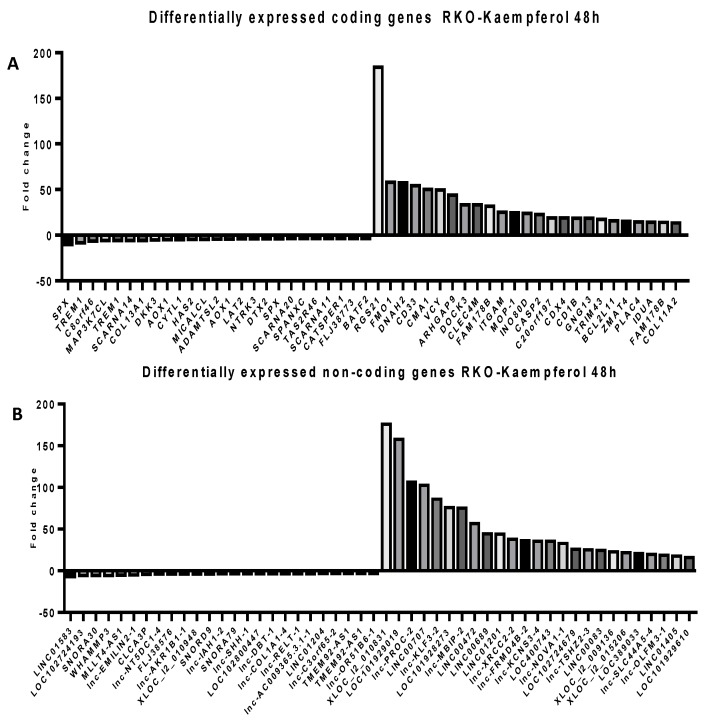
Microarray analysis in colon cancer cells after treatment with Kaempherol. Differential expressed (**A**) coding and (**B**) noncoding genes in RKO cells treated with 17.42 µM of Kaempherol for 48 h compared to the control counterparts that did not received any treatment.

**Figure 8:**
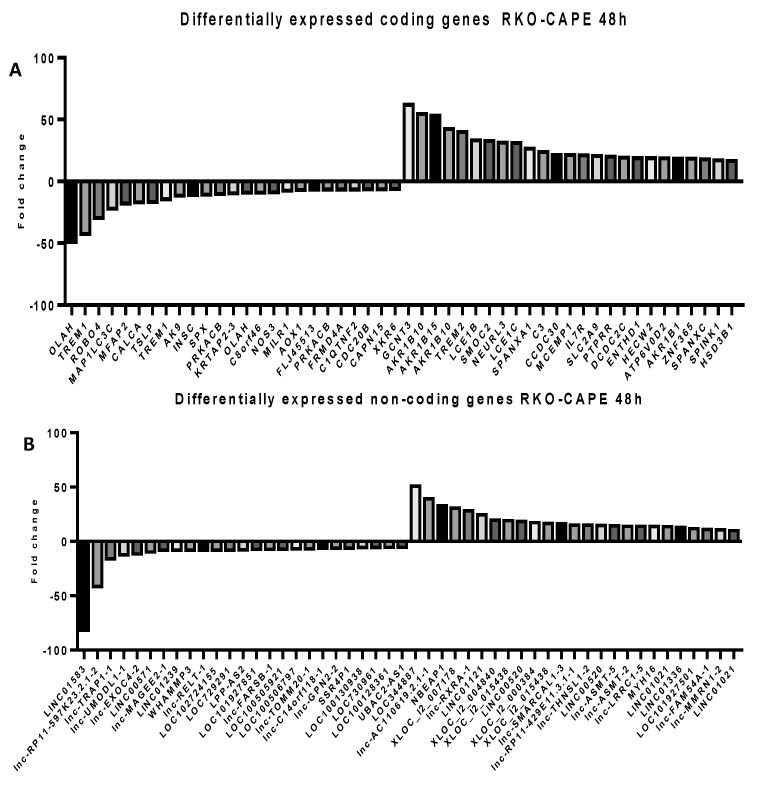
Microarray analysis in colon cancer cells after treatment with CAPE. Differential expressed (**A**) coding and (**B**) noncoding genes in RKO cells treated with 36.87 µM of CAPE for 48 h compared to the control counterparts that did not received any treatment.

**Figure 9:**
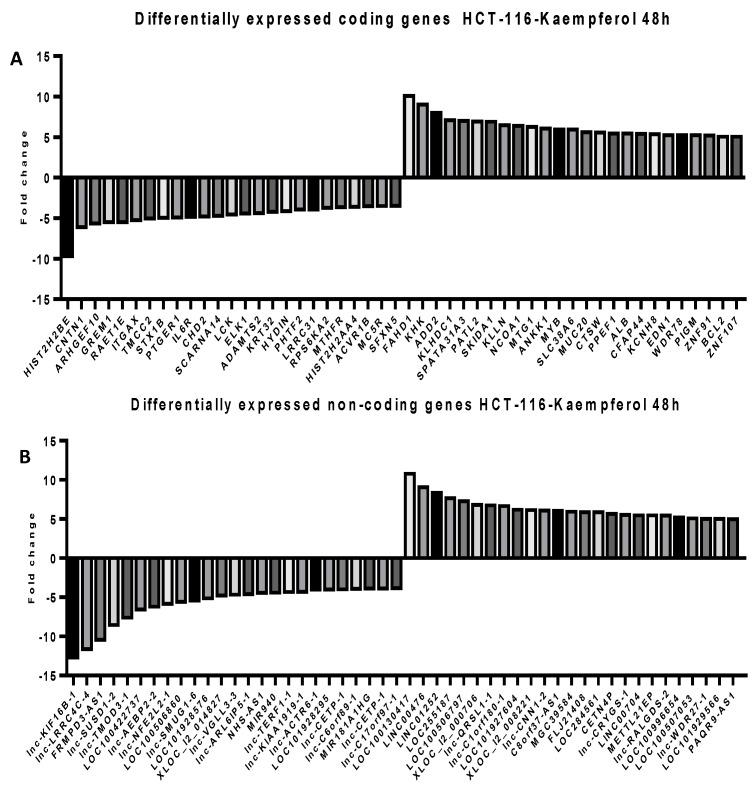
Microarray analysis in colon cancer cells after treatment with Kaempherol. Differential expressed (**A**) coding and (**B**) noncoding genes in HCT-116 cells treated with 9.427 µM of Kaempherol for 48 h compared to the control counterparts that did not received any treatment.

**Figure 10:**
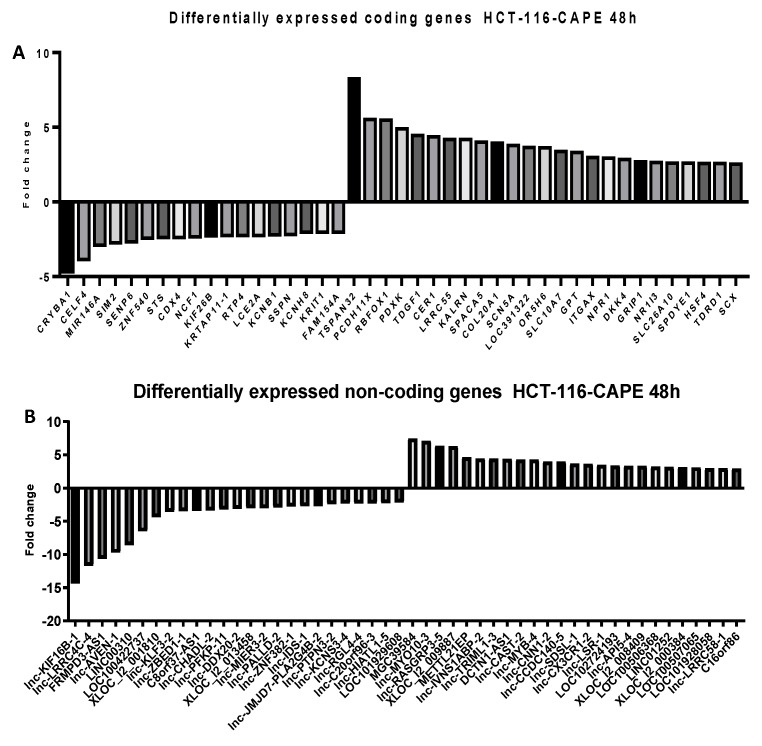
Microarray analysis in colon cancer cells after treatment with CAPE. Differential expressed (**A**) coding and (**B**) noncoding genes in HCT-116 cells treated with treated with 3.326 µM of CAPE for 48 h compared to the control counterparts that did not received any treatment.

To follow the modifications at the transcriptomic level in RKO and HCT-116 cells treated with the corresponding doses of CAPE and Kaempherol for 48 h, a microarray analysis was employed for both coding and noncoding genes. We selected to investigate the expression of long non-coding RNAs (lncRNAs) from the noncoding group, sequences with wide implications in the installation and development of cancer [24,25,26], but insufficiently explored at a global level in connection with natural compounds’ effects [8]. Specifically, we selected the top 25 most upregulated and top 25 most downregulated coding and noncoding genes in comparison with the control cells that did not receive any type of treatment (Supplementary Table S1—RKO/Kaempherol/coding; Supplementary Table S2—RKO/Kaempherol/non-coding, Supplementary Table S3—RKO/CAPE/coding, Supplementary Table S4—RKO/CAPE/non-coding, Supplementary Table S5—HCT-116/Kaempherol/coding, Supplementary Table S6—HCT-116/Kaempherol/non-coding, Supplementary Table S7—HCT-116/CAPE/coding, Supplementary Table S8—HCT-116/CAPE/non-coding). The RKO cell line treated with Kaempferol (Figure 7) and with CAPE (Figure 8) presented a trend toward upregulation of both specific coding and noncoding genes, while minimal differences were observed in the group of downregulated ones. For HCT-116 treated with Kaempferol (Figure 9) and with CAPE (Figure 10), the transcriptome was modified toward both upregulation and downregulation of coding and noncoding genes. Moreover, the pattern of the top 25 modified genes is different between cell lines treated with the same compound and within the same cell line exposed to CAPE or Kaempherol, demonstrating the specific value of natural compounds and particular altered pathways. Besides, there is also the possibility that the same gene is differentially regulated according to the specific treatment; this is the case of KCNH8 (Potassium Voltage-Gated Channel Subfamily H Member 8) that is downregulated in HCT-116 treated with CAPE and upregulated in the same cell line treated with Kaempherol. 

After analysing the entire coding transcriptomic profile using Panther software for generation of Biological Processes for colon cancer cells treated with CAPE and Kaempherol, we observed the main mechanisms which are altered upon the activity of these natural compounds. Kaempherol affects in RKO cell line: cellular component and organisation or biogenesis, immune system, and metabolic processes and response to stimulus, these pathways being common for both downregulated and upregulated genes (Supplementary Figure S2A). Moreover, the upregulated genes are also interfering with biological adhesion with possible influence upon cell invasion (Supplementary Figure S2A). In the same cell line, CAPE interferes with the same pathways for both downregulated and upregulated genes; although, this compound also has roles in modulation of cell killing (for upregulated genes) and rhythmic process (for both upregulated and downregulated genes) (Supplementary Figure S2B). Rhythmic process has been connected with cell cycle and cancer development, although much effort has to be attributed in this specific area of study [27]. For both treatments and cell lines, the interference with metabolic processes is one of the principal triggers (Supplementary Figure S2). HCT-116 cell line treated with Kaempherol is modulated in terms of biological adhesion, cellular component organisation or biogenesis, developmental processes, immune system, and metabolic processes and response to stimulus regarding both downregulated and upregulated genes; these pathways are similar with the ones affected in the previous cell line. Regarding only the downregulated profile of genes, Kaempherol also interferes with cell killing with possible roles in induction of apoptosis (Supplementary Figure S3A). CAPE has similar effects and controls cellular component and organisation or biogenesis, developmental and metabolic processes (Supplementary Figure S3B). Also, the alteration in metabolic pathways is one of the most pronounced effects for both treatments. 

In order to deepen the mechanistic insights upon the modifications from the transcriptional level, we analysed the biological processes (Panther software) modulated by the top 25 downregulated and upregulated respective coding genes for both cell lines and treatments (Supplementary Figures S4–S7). Exposure of the RKO cell line to Kaempherol (Supplementary Figure S4) upregulates the expression of CASP2 that is involved in cellular component organisation or biogenesis, cellular processes, biological regulation, and also metabolic processes. This gene has been assigned with a tumour suppressor role in cancer, a role that is connected to cell death but also with cellular stress and transformation; moreover, loss of CASP2 promotes carcinogenesis in in vivo models of malignant pathologies [28]. Another gene found repeatedly in different biological processes and upregulated after Kaempherol treatment is CLEC4M, also known as DC-SIGNR according *NCBI-Gene* (Gene ID: 10332). CLEC4M is involved in localisation, biological regulation, response to stimulus, biological adhesion, and immune system processes according to our analysis. Despite minimal data regarding the role of this gene in cancer, a recent study demonstrated that CLEC4M actually supports adherence, invasion, and liver metastasis in colorectal cancer with minimal difference upon cell proliferation [29]. Gene profiling in colorectal cancer cells incubated with DC-SIGNR protein showed that the effects of this molecule is majorly mediated by metallothionein family members with no differences in genes connected to liver metastasis (e.g., MET, SMAD7, or DRG1). After comparing our gene profile from RKO treated with Kaempherol and the data from the reminded study, we found no increase or decrease in metallothioneins, except MT1IP that was not mentioned in the previous research (data not shown). Furthermore, no similarity between the gene profiles was seen, except for the upregulation of MMP28 (data not shown). In terms of downregulated genes after Kaempherol treatment in RKO, we found that NTRK3 is associated with multiple biological processes (cellular component organization or biogenesis, cellular processes, biological regulation, response to stimulus, developmental process) and is also inhibited by exposure to the natural compound. NTRK3, also known as TrkC, was found as a dependency receptor in colorectal cancer where its tumour suppressor or oncogenic function is dependent on the expression of its ligand NT-3; specifically, the receptor–ligand interaction promotes cell survival, but in the NT-3-free form, NTRK3 becomes a tumour suppressor by inducing apoptosis [30]. In our case, we found no significant difference in the expression of NT-3 (data not shown) (the expression of NT-3 remains to be established in normal and malignant colon tissue). The downregulated profile of NTRK3 together with the functional in vitro test could possibly sustain a tumour-promoting role of this gene. This affirmation is confirmed in a more recent study where TrkC was associated with increase in colorectal cancer growth, formation of spheroids, and invasion [31]. Treatment of the RKO cell line with CAPE (Supplementary Figure S5) showed different modulated genes, but similar affected mechanisms. Specifically, we identified TREM2 as upregulated and involved in the most pronounced (in terms of percentage) biological processes: localization and response to stimulus. Despite minimal data for colon cancer, TREM2 was recently associated with a tumour suppressor role in hepatocellular carcinoma by decreasing metastasis through interaction with epithelial to mesenchymal transition (EMT) [32]. In the case of downregulated genes, XKR6 showed wide implications in different biological processes. However, the role of this gene remains to be determined in colorectal cancer. According to UniProt (UniProtKB -Q5GH73-XKR6_HUMAN), XKR6 is connected with apoptotic processes involved in development and is expressed in 7 out of 12 patients with colorectal malignancies (data from The Human Protein Atlas).

The same two treatments showed different results in terms of the top 25 downregulated and upregulated genes in the second cell line—HCT-116. For Kaempherol (Supplementary Figure S6), within the list of upregulated genes, one of the most widely involved in different biological processes is Bcl-2. Despite the fact that this gene is generally associated with an oncogenic role and is used as a therapeutic target, in colorectal cancer the increased expression of Bcl-2 is actually a good prognostic factor according to a recent metaanalysis [33]. Further data should revel if the activity of Kaempherol is Bcl-2 specific or is an effect specific for colon cancer inhibition. Bcl-2 is also upregulated in the RKO cell line after treatment with Kaempherol (data not shown). The inhibitory roles of Kaempherol in HCT-116 cells are sustained also through downregulation of IL6R, which is part of multiple biological processes, including biological regulation, response to stimulus, and biological adhesion. The oncogenic role of IL6R in mediation of colorectal tumour invasion via activation of EMT was demonstrated though modulation of the IL-6R/STAT3/miR-34a feedback loop [34]. Treatment of the same cell line with CAPE (Supplementary Figure S7) showed different modulated genes within the top 25 upregulated and downregulated ones. The most prominent was CER1, gene found across multiple biological processes and upregulated in response to in vitro CAPE administration. The role of this gene is minimally explored in cancer, except for some evidence of preferential loss in human tumours [35]. 

### 2.6. Next Generation Sequencing in RKO and HCT-116 Cell Lines Treated with Cape and Kaempherol

To investigate the depth of polyphenols activity, RKO and HCT-116 cell lines were sequenced at the DNA level after 48 h of exposure to Cape and Kaempherol in comparison with control cells that did not receive any type of treatment (row data in Supplementary Table S9). In RKO cells, both Cape and Kaempherol determined the origins of three new mutations: CSFR1 c.*37delA, c.*35insT (c.* is for mutations in the untranslated regions of a gene), and TP53 c.215C>G, but abolished the presence of the c.3196G>A mutation from the PIK3CA gene level. This last mutation is associated with pathogenic roles according to Functional Analysis through Hidden Markov Models (FATHMM) score from Cosmic. The TP53 c.215C>G is a benign mutation, according to FATHMM score, and it appears to be connected to drug response in ClinVar. The other two mutations are new mutation that have no clinical characterization yet. 

**Figure 11:**
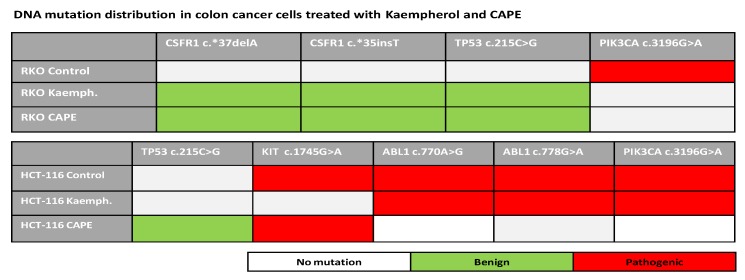
Next generation sequencing in RKO and HCT-116 cell lines treated with Cape and Kaempherol. RKO cells without treatment (Control) and treated with 36.87 µM of CAPE and 17.42 µM of Kaempherol for 48 h; HCT-116 cells without treatment (Control) and treated with 3.326 µM of CAPE and 9.427 µM of Kaempherol for 48 h. White colour shows the lack of mutation, whereas green and red indicate the presence of benign and pathogenic mutations, respectively. Mutations in the untranslated regions (UTR) of a gene have the coding c.*.

In the HCT-116 cell line, treatment with Kaempherol abolished the presence of KIT c.1745G>A, which is considered pathogenic according to FATHMM score from Cosmic, and did not induce the acquisition of the TP53 c.215C>G mutation. For CAPE, the mutational pattern is different in HCT-116 with introduction of TP53 c.215C>G mutation and abolishment of ABL1 c.770A>G, ABL1 c.778G>A and PIK3CA c.3196G>A, two of them being associated with a pathogenic role by FATHMM score from Cosmic, and the ATM c.778G>A being a new discovery for which there is no clinical characterization (Figure 11). 

The modifications of the DNA landscape after treatment with CAPE and Kaempherol show the potential of natural compounds to act deep at molecular level. However, it remains to be elucidated if these modifications are controlled or represent a random modulation possibly caused by changes in the transcriptomic profile with roles in DNA repair. 

## 3. Discussion

Natural compounds are known for their beneficial effect in the health sector; however, the development in bench research and clinical investigation has revealed that these compounds act deeply at the molecular level with controlled anticancer activities [8,36]. 

CAPE is a natural phytochemical compound derived from propolis, which has many biologically active properties, such as: antibacterial, antioxidant, antiviral, and anti-inflammatory activity [15,16,17]. Kaempferol is a natural flavonoid found in a variety of plants and plant derivatives, such as: apples, onion, leeks, citrus, grapes, gingko biloba, St. John’s wort, red wine, and others. This compound is a powerful antioxidant agent that also inhibits the formation of malignant cells [19,20,21,22].

In the present study, we investigated the in vitro role of natural compounds in colon cancer inhibition, one of the top-ranking cancers in terms of incidence and mortality. Specifically, we tested the activity of CAPE, Kaempferol, Morin, EGCG, Daidzein, and Genistein upon cell viability and we further continued with functional test for CAPE, Kaempferol, and Morin. Finally, CAPE and Kaempferol were used for analysis of gene (coding and non-coding) expression in treated cells compared to control ones. 

All compounds tested had inhibitory effects in terms of cell viability, but also in dose dependency. Following the MTT cell viability test, the IC_50_ (half maximal inhibitory concentration) obtained for CAPE on RKO cell line is 36.87 μM and on HCT-116 is 3.326 μM, lower concentrations compared to those previously obtained by Hao Tang et al. [37] on the HCT-116 cell line (IC_50_ = 47.2 μM). Evaluation of cell cycle showed that natural compounds have the ability of stopping the cell cycle with a preponderance of the treated cell population in G1 phase compared to control samples. Morin exhibited the most pronounced effects on the RKO cell line, while CAPE had the strongest effect upon cell cycle in HCT-116 cells. In terms of cell invasion, CAPE treatment limited the migratory parameters in treated cells compared with control; minimal effects were observed for the other two natural compounds, Kaempferol and Morin. These results are in concordance with the previous study of Wu et al. [38] where treatment with CAPE on colon cancer cell lines (different cell line: SW-480) resulted in a significant decrease (*p* < 0.05) of both cell motility and invasion.

Microscopic evaluation has confirmed the ability of CAPE and Kaempferol in inducing cell apoptosis and cellular disorganisation, showing a high degree of disrupted cells with direct effects also upon their number. 

Microarray data for coding and noncoding genes reveal a differential transcriptomic pattern between control and treated cells. Analysis of the biological processes generated after inclusion of the upregulated and downregulated coding genes show that CAPE and Kaempferol are generally modifying pathways related to biological adhesion, cellular component and organisation or biogenesis, developmental, immune system and metabolic processes, response to stimulus, and cell killing. 

From NGS data, the RKO lines treated with CAPE and Kaempferol show some mutations in addition to the control sample: CSFR1 c.*37delA, c.*35insT, and TP53 c.215C> G, which are considered benign but importantly, do not exhibit the mutation c.3196G>A from the PIK3CA gene level, which is considered malignant. HCT-116 control lines have pathogenic mutations: KIT c.1745G>A, ABL1 c.770A>G, ABL1 c.778G>A and PIK3CA c.3196G>A, whereas those treated are associated only with acquisition of benign mutations such as: TP53 c.215C>G in the case of CAPE treatment and partial loss of the pathogenic ones (differential results for CAPE and Kaempferol). These data show the in vitro potential of natural compounds to act deeply at the molecular level affecting the structure of DNA toward cancer inhibition. 

## 4. Materials and Methods 

### 4.1. Cell Lines 

Colon cancer cell lines RKO, HCT-116 and HT-29 and DLD-1 (American Type Culture Collection, Manassas, VA, USA) were cultivated in McCoy’s medium supplemented with 10% fetal bovine serum (FBS), 100 UI/mL penicillin and 100 µg/mL streptomycin (for RKO, HCT-116 and HT-29) and RPMI-1640 medium supplemented with 10% FBS, 2mM l-Glutamine and 100 UI/mL penicillin and 100 µg/mL streptomycin (for DLD-1). Adherent cells were maintained at 37 °C in 5% CO_2_ atmosphere and 95% humidity in 25 cm^2^ and 75 cm^2^ culture flasks. Using an optical microscope, we regularly monitored the morphological appearance, differentiation level and cell density. Cell splitting was performed after the monolayer has reached a confluence of 80–90%. 

### 4.2. Cellular Treatment

Cells were treated for 48 h with phytochemical compounds (CAPE, Kaempferol, Morin, EGCG, Daidzein and Genistein) of different concentrations: 0.1; 1; 10; 50; 100; 250; 500; 1000 μM.

### 4.3. MTT (3-(4,5-Dimethylthiazolyl-2)-2,5-diphenyltetrazolium bromide) Assay

A number of 1 × 10^4^ cells/well was seeded in the 96-well plate in a volume of 200 μL and incubated for 24 h at 37 °C. After 24 h the cells were treated with solutions of phytochemical compounds of different concentrations. MTT was performed 48 h after treatment. The culture medium was removed and the cells were suspended in 150 μL of 1 mg/mL MTT solution (Sigma, St. Louis, MO, USA). After 1 h of incubation at 37 °C, the formazan crystals were solubilized in 100 μL of DMSO and then the absorbance was read at 570 nm and 690 nm using a Biotek Synergy HT microplate reader (Winooski, VT, USA).

### 4.4. Cell Cycle Evaluation 

Cells detached after trypsinization were collected, washed with PBS 1× and then fixed for 45 min at 4 °C with 70% ethanol, which was previously cooled to −20 °C. After fixation, the cells were washed again with PBS 1×, which was then completely discarded. The cell pellet was resuspended in RNAase buffer (0.2% BSA salt with 40 μg/mL RNAse A, the solvent being PBS 1×) and incubated for 15 min at room temperature in the dark. PI was added to this mixture, resulting in a pale pink final coloration. The flow cytometry (BD FACS Canto II instrument, San Jose, CA, USA) analysis was performed after incubation for 30 min at room temperature in the dark and the interpretation of the results was done with FACS Diva software version 6.0 (San Jose, CA, USA). 

### 4.5. Evaluation of Cell Invasion 

150,000 cells/well were seeded and after 24 h, RKO and HCT-116 cell lines were treated with 100 μM solutions of CAPE, Morin, and Kaempferol. After 48 h mitomycin was added, a reagent that brings cells into the same phase of the cell cycle. The bottom of the well was scratched with a 20 μL pipette tip and the medium was removed and replaced with a new one. The cells were monitored and images were captured at 0, 24, 48, 72, and 96 h. Pictures were taken with Olympus IX71 microscope (Shinjuku-ku, Tokyo, Japan) using cellSens software (scale bar—200 µM). Resulted images were processed with ImageJ Software (version 2.0, Madison, WI, USA) in order to calculate the percent of area free of cells in treatment groups compared to control ones. 

### 4.6. Apoptosis Assay

Cells were cultured on 96-well plates overnight at 37 °C and then treated and incubated for 24/48 h at 37 °C, 5% CO_2_. One hundred μL of TMRE/Hoechst solution was added and after 10–30 min incubation at 37 °C the cells were centrifuged at 400× *g* for 5 min. The supernatant was discarded and the cells were washed with PBS 1× and examined with 560/595 nm fluorescence microscope (Olympus, Shinjuku-ku, Tokyo, Japan) for TMRE and UV for Hoechst.

### 4.7. Autophagy Detection

Cells were cultured on 96-well plates overnight at 37 °C and then the treatment was performed. The cells were incubated for 24/48 h at 37 °C, 5% CO_2_. After 400 *g* centrifugation the supernatant was discarded and 100 μL of propidium iodide (PI) solution was added. After a 10 min incubation period at room temperature, the supernatant was discarded and washed with PBS 1×. 100 μL MDC solution (monodansilcadaverine) was added. After 10 min at 37 °C incubation, the supernatant was discarded and washed with PBS 1×. For examination of the cells it was used a fluorescence microscope (Olympus, Shinjuku-ku, Tokyo, Japan).

### 4.8. Dark-Field Microscopy

Was performed on an inverted Olympus BX43 microscope (Olympus, Tokyo, Japan) equipped with a CytoViva Enhanced Dark-Field Condenser (CytoViva, Auburn, AL, USA), UPlanFLN60X, NA = 1.2 oil immersion objective and a dual-fluorescence module. Emission and excitation lights were filtered through a triple-pass (DAPI/FITC/Texas Red) filter.

### 4.9. Confocal Microscopy

Laser scanning confocal microscopy was performed on an Olympus FV1200 (Olympus, Tokyo, Japan) inverted confocal microscope. Excitation and emission wavelengths were autoselected by the acquisition software (FV10-ASW 4.2) from a fluorophore repository. The objective used was a UPLSAPO40X2, NA = 0.95. Imaging was performed in line sequential mode to prevent bleed-through.

Image calibration and processing for all image sets was performed in Image J version 2.0 (https://imagej.nih.gov/ij). Calibration was performed according to individual image metadata. Processing involved low tolerance bandpass filtering for DIC images and brightness and contrast adjustment based on the intensity distribution histogram for each channel individually. 

### 4.10. Microarray Assay

The microarray probes were synthesized from equal quantities for each sample (200 ng of total RNA), by using Agilent Low Input Quick Amp Labeling Kit (Santa Clara, CA, USA, 5190-2305) according to the manufacturer’s protocol. Subsequent to this step, the hybridization products were purified using RNeasy Mini kit (Qiagen, Hilden, Germany). Probe quality control was checked using NanoDrop2000 spectrophotometer (Thermo Scientific, Wilmington, NC, USA) and hybridisation was done using 600 ng of Cy3-labelled cDNA (specific activity higher than 6 pmol Cy3/ug cDNA). The fragmentation and hybridization were performed based on the Agilent one color protocol. The samples were hybridized (17 h at 65 °C) on a custom microarray slide using SureDesign (Agilent, Santa Clara, CA, USA). The microarray slides were scanned with the SureScan Microarray Scanner (8 × 60 k array slides with 61 × 21 mm, resolution 3 μM) from Agilent and the images were processed with Feature Extraction 11.0.1.1 software (https://www.agilent.com/home/more-countries?currPageURL=http://www.agilent.com/en/product/mirna-microarray-platform/mirna-microarray-software/feature-extraction-software-228496). GeneSpring GX v.13.0 (http://genespring-support.com) from Agilent was used for data analysis, which started with the removal of control probes and normalization as previously explained [39].

### 4.11. NGS Assay—DNA Sequencing 

Twenty ng of DNA were used for sequencing using the Ion AmpliSeq Cancer Hotspot Panel v2 (ThermoFischer Scientific, Waltham, MA, USA) and the Ion AmpliSeq Library 2.0 kit (Thermofisher Scientific, Waltham, MA, USA). The Ion AmpliSeq Cancer Hotspot Panel v2 consists of primers for hotspot evaluation in the following genes: *ABL1, AKT1, ALK, APC, ATM, BRAF, CDH1, CDK2A, CSF1R, CTNNB1, EGFR, ERBB2, ERBB4, EZH2, FBXW7, FGFR1, FGFR2, FGFR3, FLT3, GNA11, GNAQ, GNAS, HNF1A, IDH1, IDH2, JAK2, JAK3, KDR, KIT, KRAS, MET, MLH1, MPL, NOTCH1, NPM1, NRAS, PDGFRA, PIK3CA, PTEN, PTPN11, RB1, RET, SMAD4, SMARB1, SMO, STK11, TP53, VHL*. After library preparation, the samples were purified using the AMpure XP Beads (Bechman Coulter, Brea, CA, USA). The purified libraries were quantified using the fluorometer Qubit 2.0 (ThermoFischer Scientific, Waltham, MA, USA) and the Qubit HS DNA kit (ThermoFischer Scientific, Waltham, MA, USA). For template synthesis, libraries were diluted to 100 pM and multiplex 4 libraries on an Ion 316 Chip (ThermoFischer Scientific, Waltham, MA, USA). The sequencing process was performed on the Ion Torrent PGM Machine (ThermoFischer Scientific, Waltham, MA, USA) using the Ion PGM HI-Q Sequencing 200 kit (ThermoFischer Scientific, Waltham, MA, USA). The data obtained after sequencing were analyzed using the Torrent Suit 5.6 (ThermoFischer Scientific, Waltham, MA, USA) and Ion Reporter 5.6 software (ThermoFischer Scientific, Waltham, MA, USA) for data trimming, alignment and variant calling. The obtained variants were filtered using the following conditions: *p* value ≤ 0.05, coverage ≥ 500. 

## 5. Conclusions

Following the functional in vitro studies on CAPE and Kaempferol, it was found that the two phytochemical compounds have an inhibitory effect on RKO and HCT-116 colon cancer cell lines and could be potentially used in the future to develop new alternative therapeutic strategies or complete the conventional treatment regimens for this malignancy. Moreover, these compounds could also be used as preventive agents for the delay or inhibition of cancer establishment. However, important aspects about the in vivo effects like dose effectiveness, effects upon healthy cells, molecular alterations, bioavailability, and long-term exposure must be addressed before formulation of coherent therapeutic strategies. 

## Supplementary Materials

Supplementary materials can be found at https://www.mdpi.com/1422-0067/20/5/1199/s1.

## Abbreviations

ncRNAs	Non-coding RNAs
CAPE	Caffeic acid phenethyl ester
MDC	Monodansilcadaverine
lncRNAs EMT	Long non-coding RNAs Epithelial to mesenchymal transition
