# Supplementary material for: Inhibitory Effect of CAPE and Kaempferol in Colon Cancer Cell Lines—Possible Implications in New Therapeutic Strategies
Source: Int J Mol Sci. 2019 Mar 9;20(5):1199. doi: 10.3390/ijms20051199 (PMC6429399; doi:10.3390/ijms20051199)
Supplement: Supplementary file 1 [file ijms-20-01199-s001.zip › Supplementary Figures.pptx]

## Slide 1
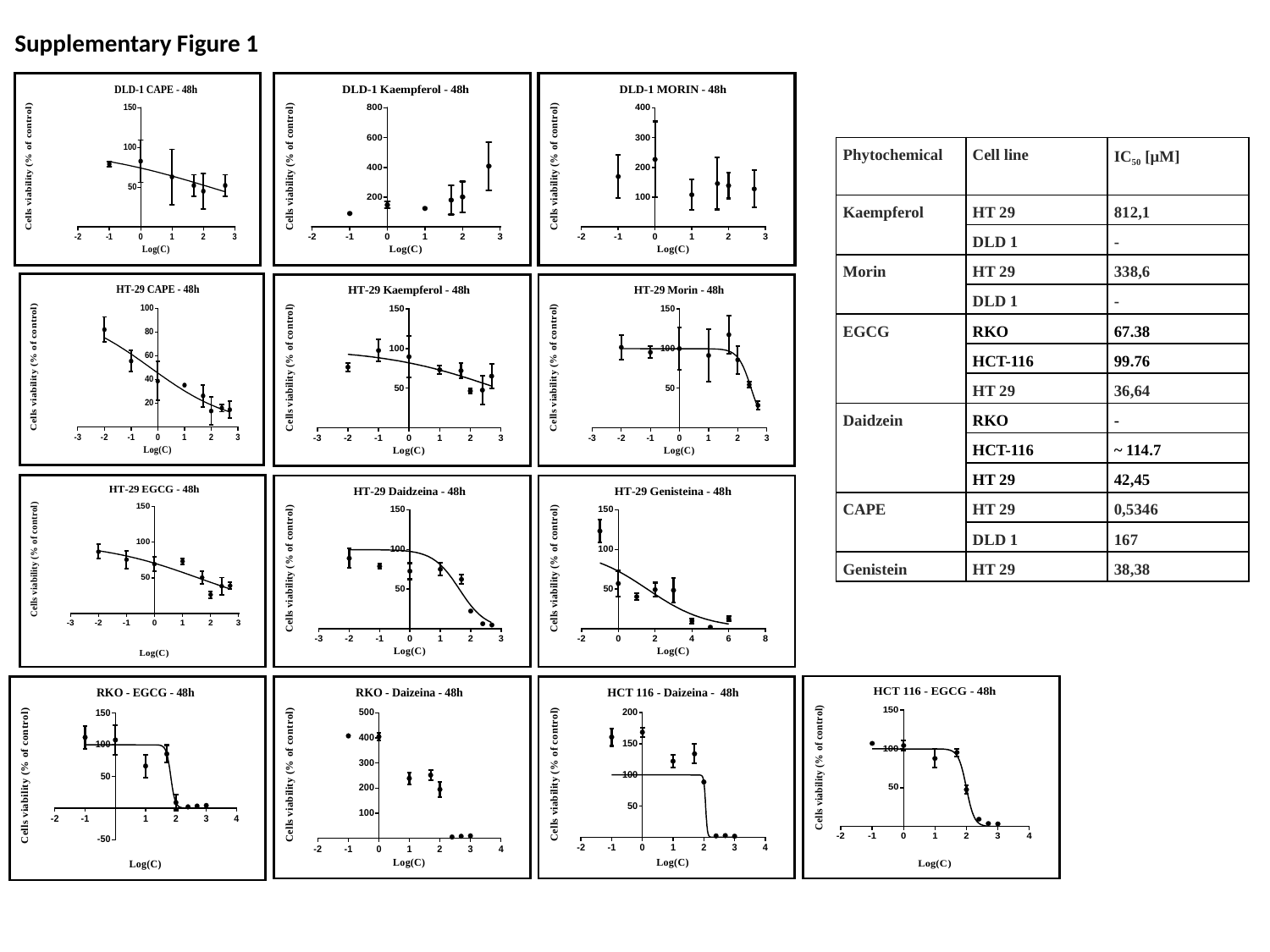

Supplementary Figure 1
| Phytochemical | Cell line | IC50 [μM] |
| --- | --- | --- |
| Kaempferol | HT 29 | 812,1 |
| | DLD 1 | - |
| Morin | HT 29 | 338,6 |
| | DLD 1 | - |
| EGCG | RKO | 67.38 |
| | HCT-116 | 99.76 |
| | HT 29 | 36,64 |
| Daidzein | RKO | - |
| | HCT-116 | ~ 114.7 |
| | HT 29 | 42,45 |
| CAPE | HT 29 | 0,5346 |
| | DLD 1 | 167 |
| Genistein | HT 29 | 38,38 |

## Slide 2
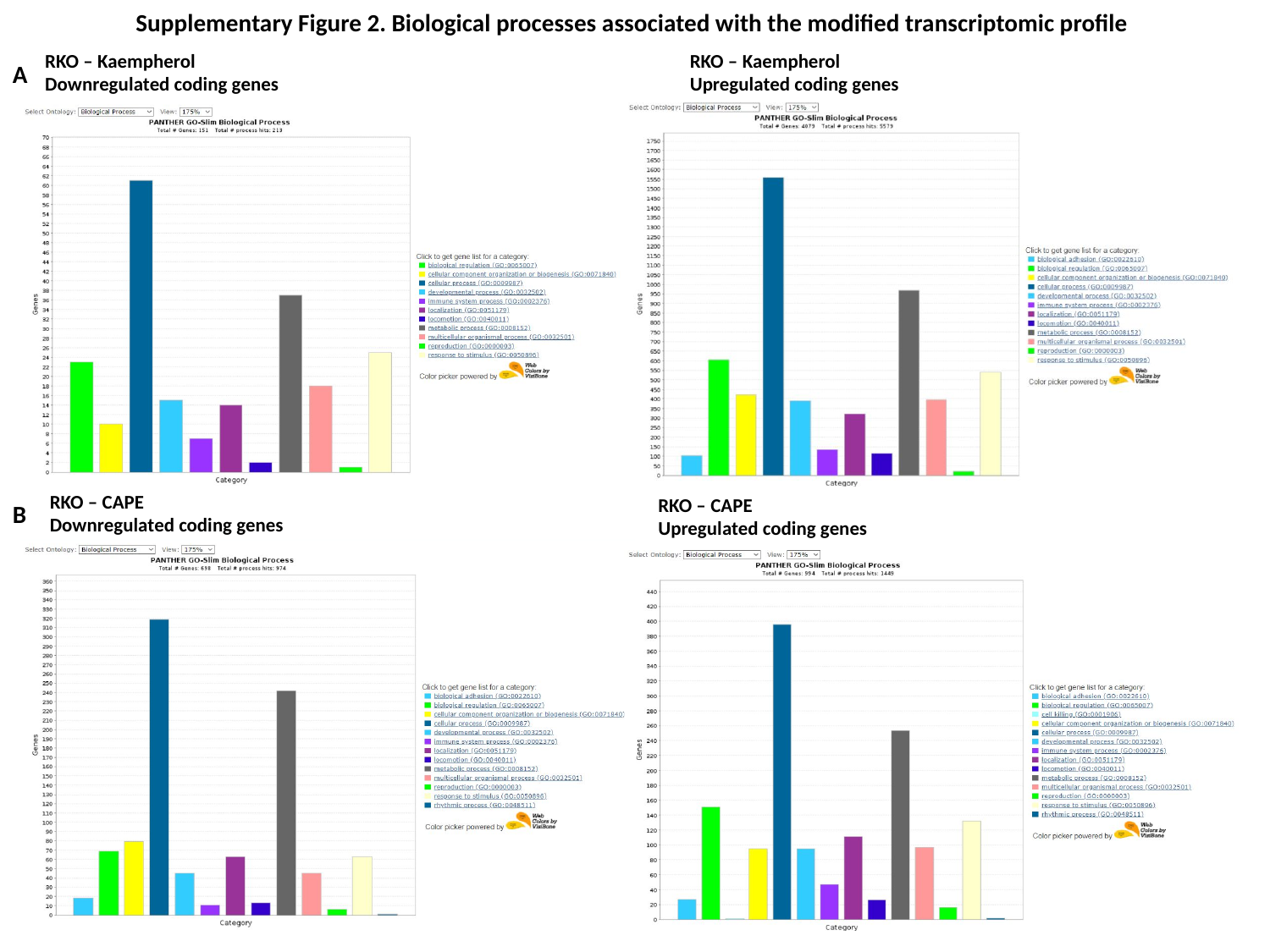

Supplementary Figure 2. Biological processes associated with the modified transcriptomic profile
RKO – Kaempherol
Downregulated coding genes
RKO – Kaempherol
Upregulated coding genes
A
RKO – CAPE
Downregulated coding genes
RKO – CAPE
Upregulated coding genes
B

## Slide 3
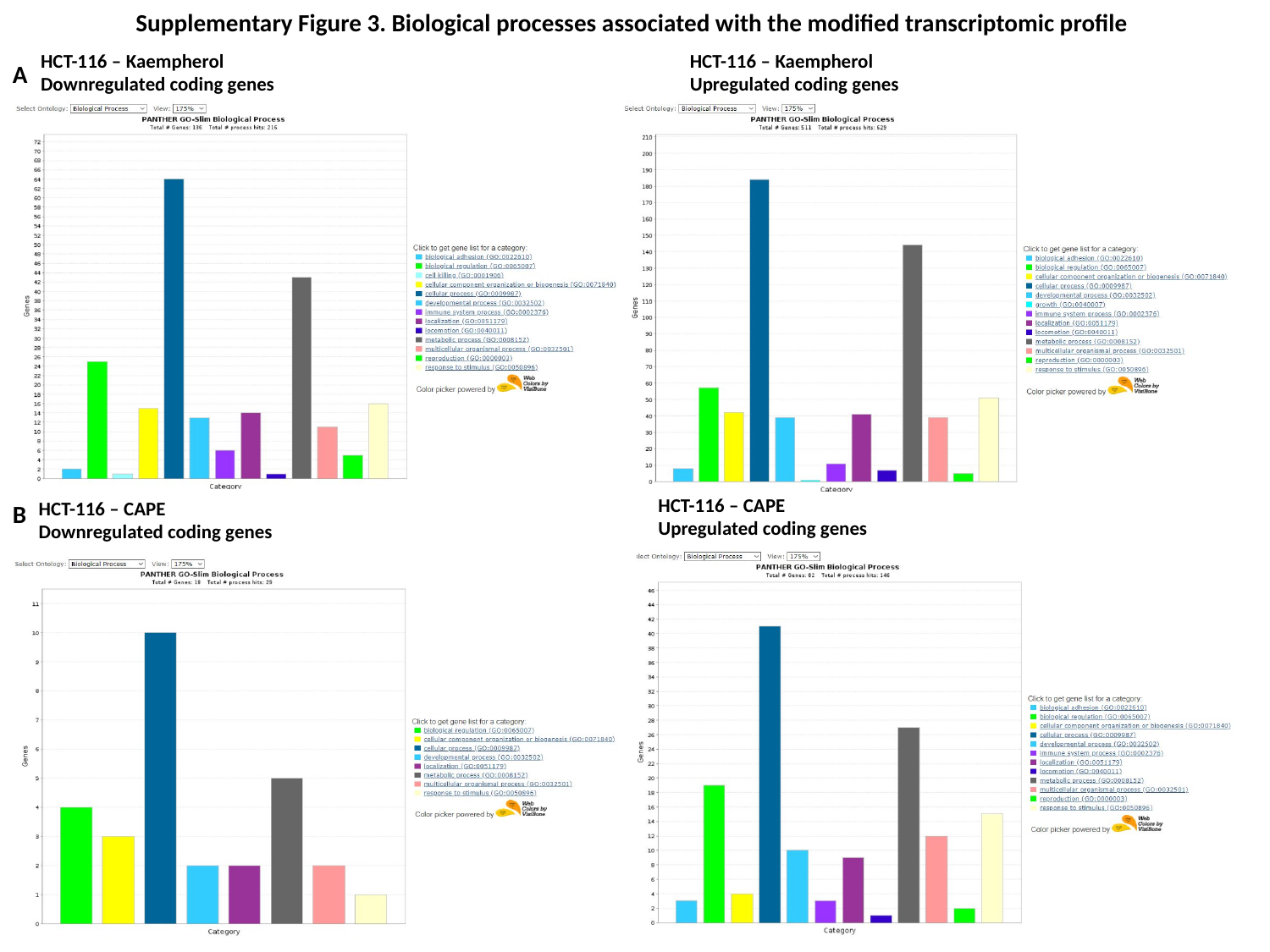

Supplementary Figure 3. Biological processes associated with the modified transcriptomic profile
HCT-116 – Kaempherol
Downregulated coding genes
HCT-116 – Kaempherol
Upregulated coding genes
A
HCT-116 – CAPE
Upregulated coding genes
HCT-116 – CAPE
Downregulated coding genes
B

## Slide 4
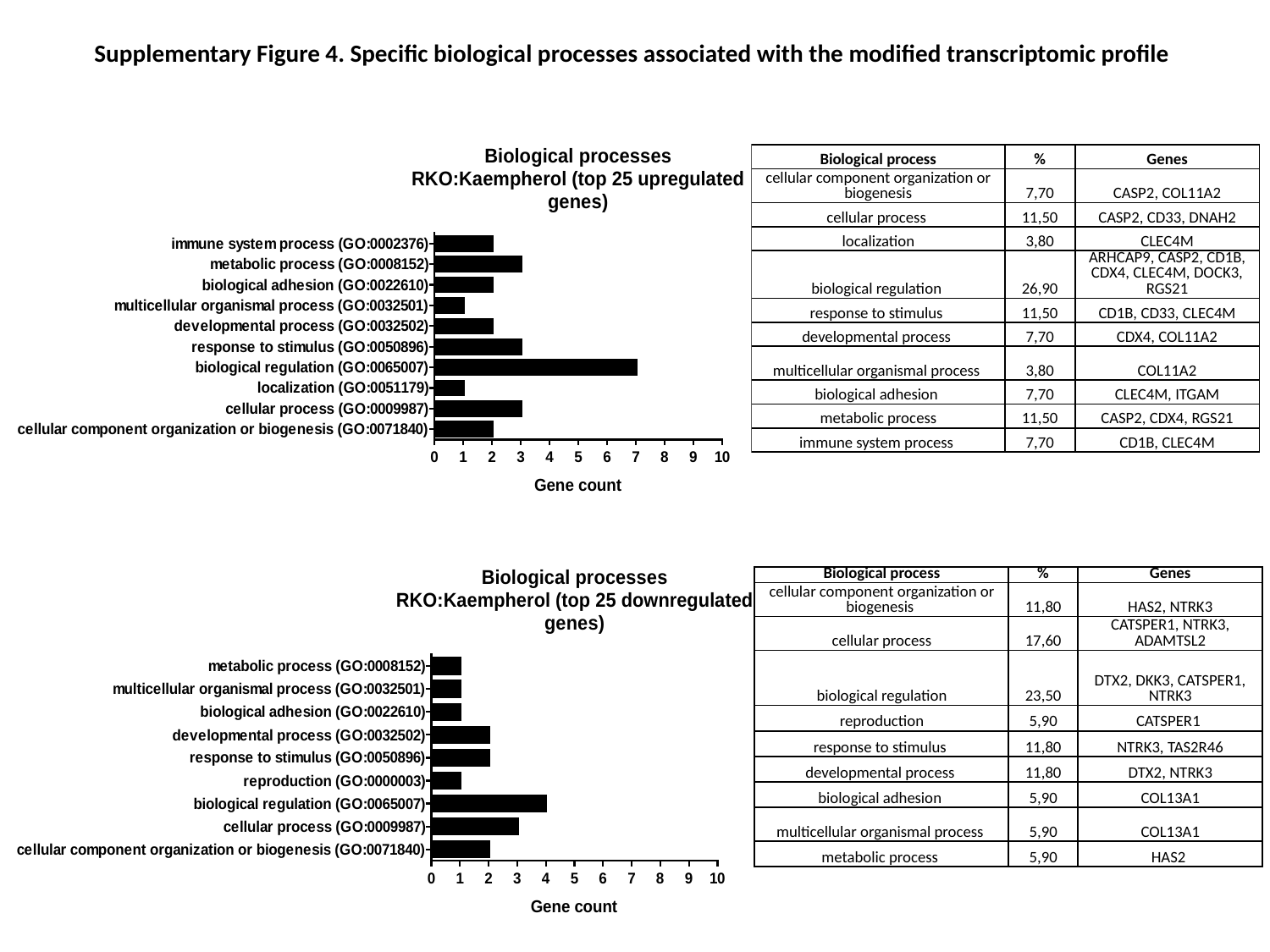

Supplementary Figure 4. Specific biological processes associated with the modified transcriptomic profile
| Biological process | % | Genes |
| --- | --- | --- |
| cellular component organization or biogenesis | 7,70 | CASP2, COL11A2 |
| cellular process | 11,50 | CASP2, CD33, DNAH2 |
| localization | 3,80 | CLEC4M |
| biological regulation | 26,90 | ARHCAP9, CASP2, CD1B, CDX4, CLEC4M, DOCK3, RGS21 |
| response to stimulus | 11,50 | CD1B, CD33, CLEC4M |
| developmental process | 7,70 | CDX4, COL11A2 |
| multicellular organismal process | 3,80 | COL11A2 |
| biological adhesion | 7,70 | CLEC4M, ITGAM |
| metabolic process | 11,50 | CASP2, CDX4, RGS21 |
| immune system process | 7,70 | CD1B, CLEC4M |
| Biological process | % | Genes |
| --- | --- | --- |
| cellular component organization or biogenesis | 11,80 | HAS2, NTRK3 |
| cellular process | 17,60 | CATSPER1, NTRK3, ADAMTSL2 |
| biological regulation | 23,50 | DTX2, DKK3, CATSPER1, NTRK3 |
| reproduction | 5,90 | CATSPER1 |
| response to stimulus | 11,80 | NTRK3, TAS2R46 |
| developmental process | 11,80 | DTX2, NTRK3 |
| biological adhesion | 5,90 | COL13A1 |
| multicellular organismal process | 5,90 | COL13A1 |
| metabolic process | 5,90 | HAS2 |

## Slide 5
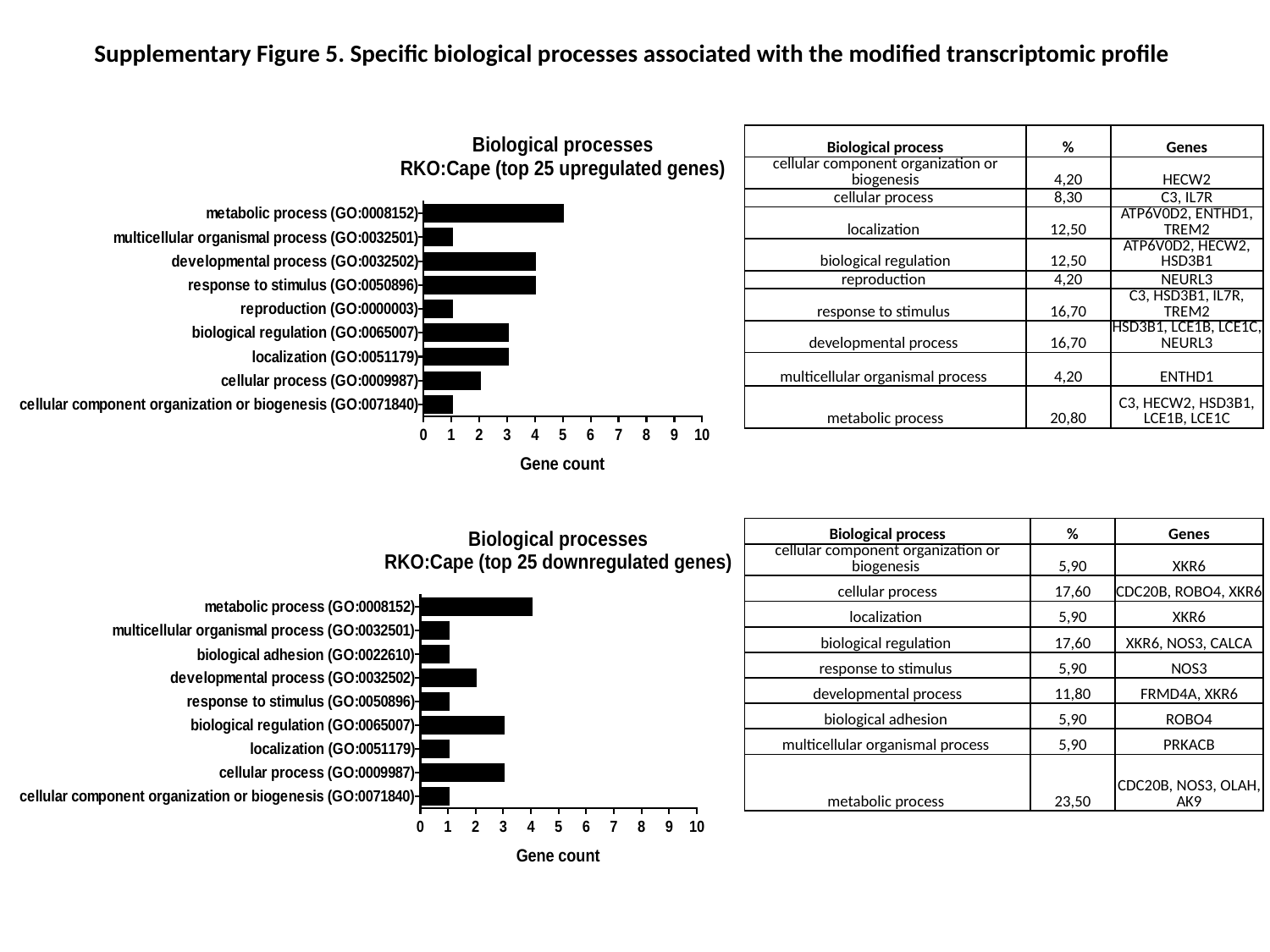

Supplementary Figure 5. Specific biological processes associated with the modified transcriptomic profile
| Biological process | % | Genes |
| --- | --- | --- |
| cellular component organization or biogenesis | 4,20 | HECW2 |
| cellular process | 8,30 | C3, IL7R |
| localization | 12,50 | ATP6V0D2, ENTHD1, TREM2 |
| biological regulation | 12,50 | ATP6V0D2, HECW2, HSD3B1 |
| reproduction | 4,20 | NEURL3 |
| response to stimulus | 16,70 | C3, HSD3B1, IL7R, TREM2 |
| developmental process | 16,70 | HSD3B1, LCE1B, LCE1C, NEURL3 |
| multicellular organismal process | 4,20 | ENTHD1 |
| metabolic process | 20,80 | C3, HECW2, HSD3B1, LCE1B, LCE1C |
| Biological process | % | Genes |
| --- | --- | --- |
| cellular component organization or biogenesis | 5,90 | XKR6 |
| cellular process | 17,60 | CDC20B, ROBO4, XKR6 |
| localization | 5,90 | XKR6 |
| biological regulation | 17,60 | XKR6, NOS3, CALCA |
| response to stimulus | 5,90 | NOS3 |
| developmental process | 11,80 | FRMD4A, XKR6 |
| biological adhesion | 5,90 | ROBO4 |
| multicellular organismal process | 5,90 | PRKACB |
| metabolic process | 23,50 | CDC20B, NOS3, OLAH, AK9 |

## Slide 6
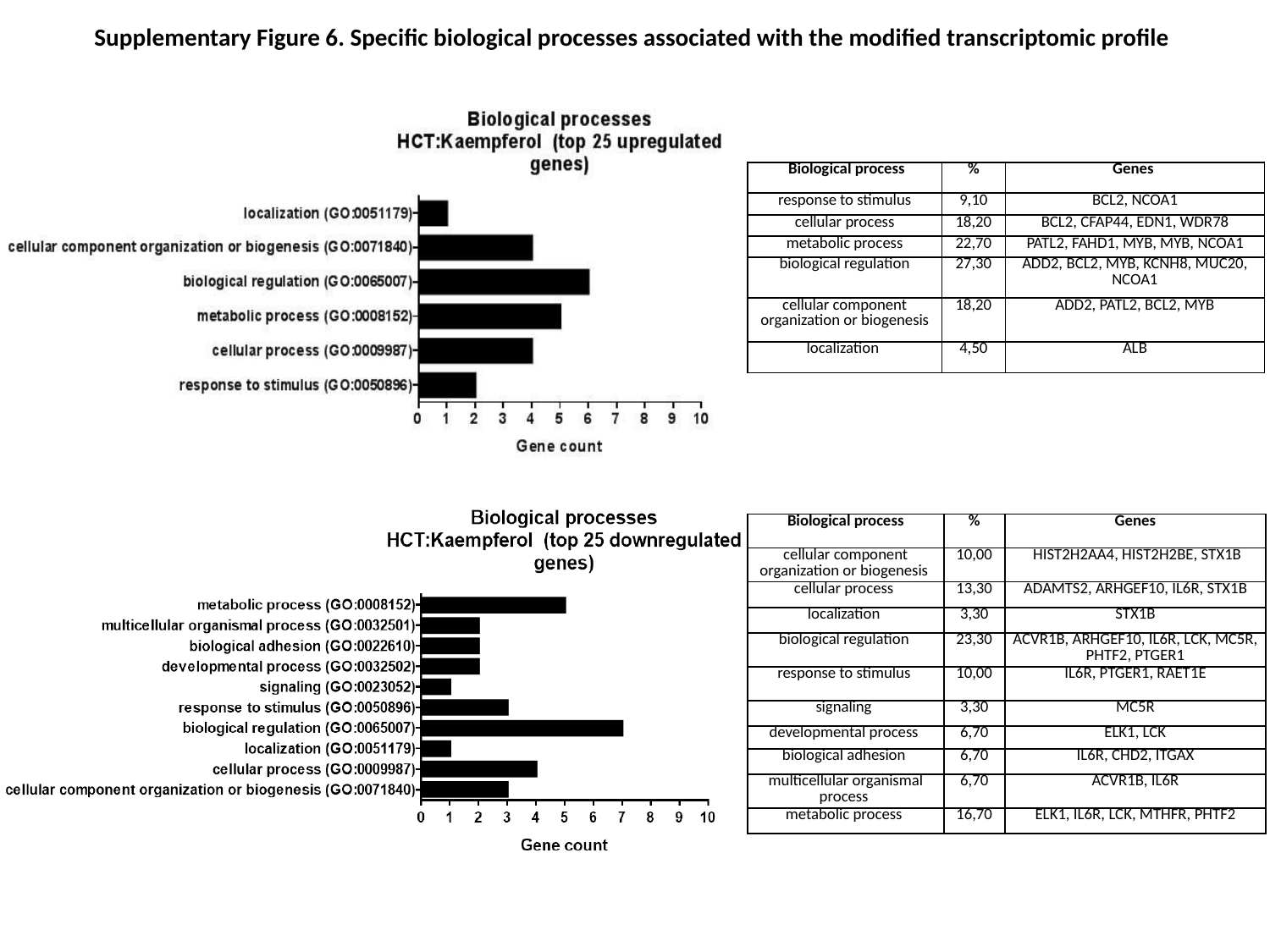

Supplementary Figure 6. Specific biological processes associated with the modified transcriptomic profile
| Biological process | % | Genes |
| --- | --- | --- |
| response to stimulus | 9,10 | BCL2, NCOA1 |
| cellular process | 18,20 | BCL2, CFAP44, EDN1, WDR78 |
| metabolic process | 22,70 | PATL2, FAHD1, MYB, MYB, NCOA1 |
| biological regulation | 27,30 | ADD2, BCL2, MYB, KCNH8, MUC20, NCOA1 |
| cellular component organization or biogenesis | 18,20 | ADD2, PATL2, BCL2, MYB |
| localization | 4,50 | ALB |
| Biological process | % | Genes |
| --- | --- | --- |
| cellular component organization or biogenesis | 10,00 | HIST2H2AA4, HIST2H2BE, STX1B |
| cellular process | 13,30 | ADAMTS2, ARHGEF10, IL6R, STX1B |
| localization | 3,30 | STX1B |
| biological regulation | 23,30 | ACVR1B, ARHGEF10, IL6R, LCK, MC5R, PHTF2, PTGER1 |
| response to stimulus | 10,00 | IL6R, PTGER1, RAET1E |
| signaling | 3,30 | MC5R |
| developmental process | 6,70 | ELK1, LCK |
| biological adhesion | 6,70 | IL6R, CHD2, ITGAX |
| multicellular organismal process | 6,70 | ACVR1B, IL6R |
| metabolic process | 16,70 | ELK1, IL6R, LCK, MTHFR, PHTF2 |

## Slide 7
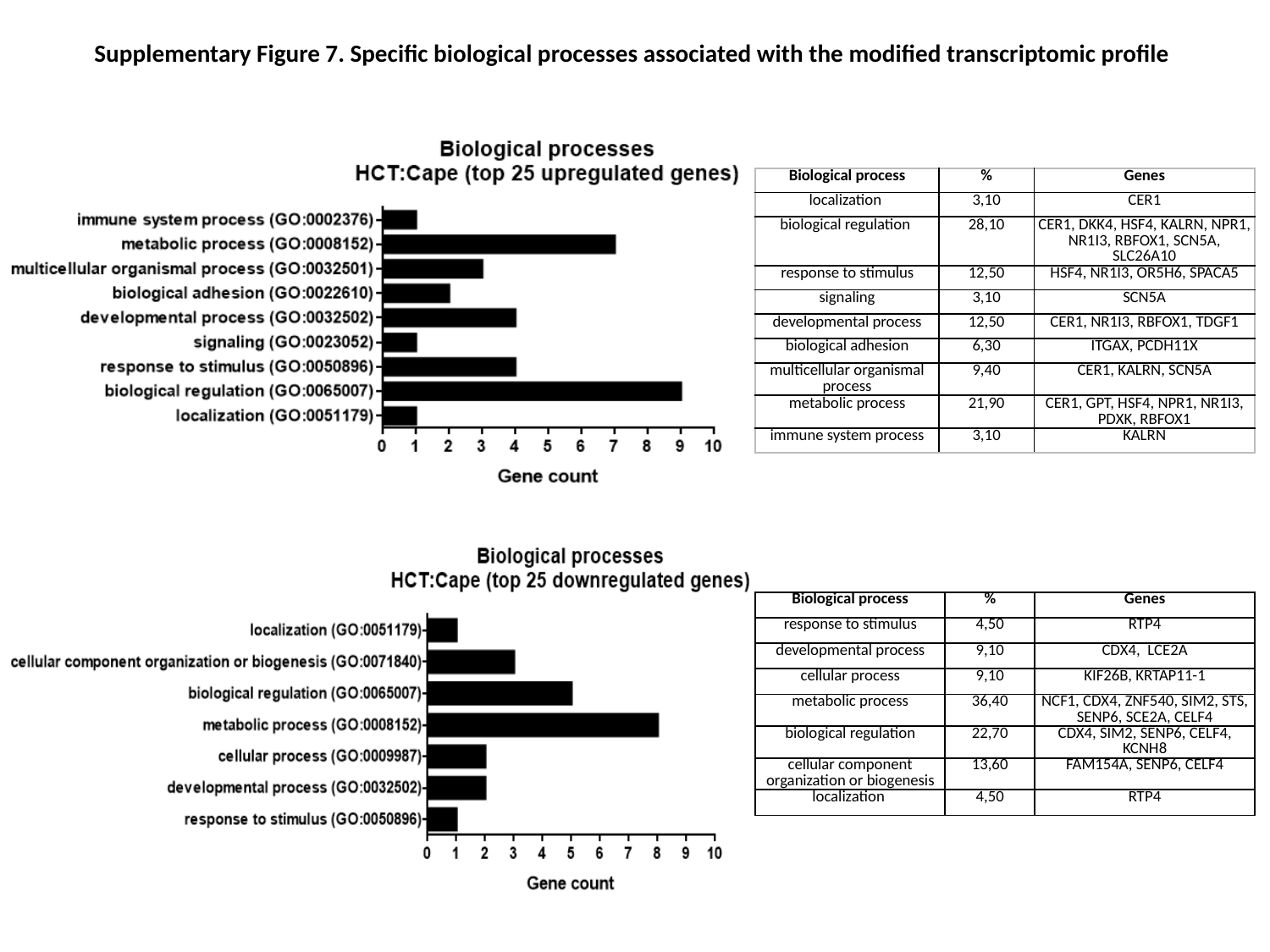

Supplementary Figure 7. Specific biological processes associated with the modified transcriptomic profile
| Biological process | % | Genes |
| --- | --- | --- |
| localization | 3,10 | CER1 |
| biological regulation | 28,10 | CER1, DKK4, HSF4, KALRN, NPR1, NR1I3, RBFOX1, SCN5A, SLC26A10 |
| response to stimulus | 12,50 | HSF4, NR1I3, OR5H6, SPACA5 |
| signaling | 3,10 | SCN5A |
| developmental process | 12,50 | CER1, NR1I3, RBFOX1, TDGF1 |
| biological adhesion | 6,30 | ITGAX, PCDH11X |
| multicellular organismal process | 9,40 | CER1, KALRN, SCN5A |
| metabolic process | 21,90 | CER1, GPT, HSF4, NPR1, NR1I3, PDXK, RBFOX1 |
| immune system process | 3,10 | KALRN |
| Biological process | % | Genes |
| --- | --- | --- |
| response to stimulus | 4,50 | RTP4 |
| developmental process | 9,10 | CDX4, LCE2A |
| cellular process | 9,10 | KIF26B, KRTAP11-1 |
| metabolic process | 36,40 | NCF1, CDX4, ZNF540, SIM2, STS, SENP6, SCE2A, CELF4 |
| biological regulation | 22,70 | CDX4, SIM2, SENP6, CELF4, KCNH8 |
| cellular component organization or biogenesis | 13,60 | FAM154A, SENP6, CELF4 |
| localization | 4,50 | RTP4 |
