# Supplementary material for: Inhibitory Effect of CAPE and Kaempferol in Colon Cancer Cell Lines—Possible Implications in New Therapeutic Strategies
Source: Int J Mol Sci. 2019 Mar 9;20(5):1199. doi: 10.3390/ijms20051199 (PMC6429399; doi:10.3390/ijms20051199)
Supplement: Supplementary file 1 [file ijms-20-01199-s001.zip › Supplementary Table 1-8.pdf]

Supplementary Table 1. Microarray results - RKO-Kaempferol 48h - The most differentially expressed coding genes (Top 25 most up/down regulated)

GeneSymbol	p (Corr)	p	Regulation	FC	Log FC
**SPX**	0,009882798	0,001819903	down	-10,126029	-3,3399966
**TREM1**	0,018556576	0,005763969	down	-8,264235	-3,0468812
**C8orf46**	0,02180454	0,007725418	down	-6,5076213	-2,7021303
**MAP3K7CL**	0,006072753	5,46E-05	down	-5,827643	-2,5429125
**TREM1**	0,014889337	0,00392886	down	-5,7947106	-2,5347366
**SCARNA14**	0,006072753	1,87E-04	down	-5,7369037	-2,5202723
**COL13A1**	0,008221181	0,001131355	down	-5,653983	-2,4992676
**DKK3**	0,010471135	0,002082346	down	-5,275981	-2,3994393
**AOX1**	0,006072753	2,91E-05	down	-4,8752646	-2,2854805
**CYTL1**	0,046108667	0,022332247	down	-4,7918663	-2,2605877
**HAS2**	0,022197437	0,007971294	down	-4,761364	-2,251375
**MICALCL**	0,006072753	6,23E-05	down	-4,393805	-2,1354709
**ADAMTSL2**	0,035923388	0,016023494	down	-4,2449026	-2,0857315
**AOX1**	0,006072753	6,70E-05	down	-4,2434382	-2,0852337
**LAT2**	0,006072753	1,35E-04	down	-4,13918	-2,049345

**NTRK3**	0,010360247	0,002029201	down	-4,1074944	-2,0382586
**DTX2**	0,033232126	0,014329448	down	-4,0376835	-2,0135279
**SPX**	0,011645762	0,002592095	down	-4,002425	-2,0008745
**SCARNA20**	0,006782571	4,48E-04	down	-3,7432098	-1,9042759
**SPANXC**	0,012453955	0,003003313	down	-3,6311977	-1,8604455
**TAS2R46**	0,019465206	0,006286474	down	-3,6034966	-1,8493974
**SCARNA11**	0,020008743	0,006529721	down	-3,5760891	-1,8383827
**CATSPER1**	0,006782571	4,74E-04	down	-3,566778	-1,8346214
**FLJ38773**	0,030505905	0,012594181	down	-3,5496218	-1,8276653
**BATF2**	0,006072753	1,29E-04	down	-3,5425966	-1,8248072
**RGS21**	0,014242152	0,003614981	up	184,0419	7,5238905
**FMO1**	0,006072753	2,71E-04	up	57,964977	5,8571095
**DNAH2**	0,006919731	6,92E-04	up	57,482327	5,8450465
**CD33**	0,006072753	2,09E-04	up	54,28184	5,762398
**CMA1**	0,021547725	0,007564163	up	50,36604	5,6543794
**VCY**	0,007443665	8,95E-04	up	49,49561	5,6292286
**ARHGAP9**	0,0187362	0,005969217	up	44,127113	5,4635935
**DOCK3**	0,007764121	0,00102234	up	33,3437	5,0593424
**CLEC4M**	0,007922231	0,001059066	up	33,24884	5,055232

**FAM178B**	0,007148277	7,70E-04	up	31,698034	4,9863214
**ITGAM**	0,006072753	1,97E-04	up	25,166553	4,6534357
**MOP-1**	0,016933363	0,004941672	up	24,58354	4,619621
**INO80D**	0,006072753	7,40E-05	up	23,891182	4,5784063
**CASP2**	0,00740808	8,76E-04	up	22,834822	4,5131636
**C20orf197**	0,006072753	9,12E-05	up	19,070473	4,2532687
**CDX4**	0,021849586	0,007772965	up	18,924606	4,2421913
**CD1B**	0,00740808	8,71E-04	up	18,703062	4,2252026
**GNG13**	0,007598146	9,63E-04	up	18,674265	4,2229795
**TRIM43**	0,007764121	0,001031146	up	17,466057	4,126482
**BCL2L11**	0,006782571	4,53E-04	up	15,7657995	3,9787264
**ZMAT4**	0,019465206	0,006292814	up	15,361171	3,9412162
**PLAC4**	0,006919731	6,90E-04	up	14,548714	3,8628197
**IDUA**	0,006072753	8,61E-06	up	14,345359	3,8425121
**FAM178B**	0,00675972	4,28E-04	up	14,116123	3,819272
**COL11A2**	0,012416313	0,002977409	up	13,479758	3,7527227

Supplementary Table 2. Microarray results - RKO- Kaempferol 48h - The most differentially expressed non-coding genes (Top 25 most up/down regulated)

GeneSymbol	p (Corr)	p	Regulation	FC	Log FC
LINC01583	0.018016826	0.005533346	down	-7.105507	-2.82894
LOC102724193	0.048313916	0.024019763	down	-5.7629976	-2.52682
SNORA30	0.018659689	0.005836757	down	-5.7235985	-2.51692
WHAMMP3	0.02450844	0.009411498	down	-5.6303387	-2.49322
MLLT4-AS1	0.025271233	0.009814821	down	-5.2181926	-2.38355
lnc-EMILIN2-1	0.006782571	6.15E-04	down	-5.0078573	-2.32419
CLCA3P	0.009724351	0.001737553	down	-4.582848	-2.19624
lnc-NT5DC1-4	0.026683021	0.010503016	down	-4.163207	-2.0577
FLJ38576	0.045130614	0.021648498	down	-4.162726	-2.05753
lnc-AKR1B1-1	0.022787007	0.00828645	down	-4.098347	-2.03504
XLOC_l2_010948	0.048241593	0.023904495	down	-4.0974116	-2.03471
SNORD9	0.014689662	0.003837666	down	-4.055664	-2.01994
lnc-IAH1-2	0.023802338	0.008880383	down	-3.8767612	-1.95485
SNORA79	0.027623486	0.010993882	down	-3.7654014	-1.9128
lnc-SHH-1	0.017477471	0.005253167	down	-3.7628505	-1.91183
LOC102800447	0.020444367	0.006885334	down	-3.732654	-1.9002
lnc-DBT-1	0.006743523	4.23E-04	down	-3.718563	-1.89475
lnc-COL1A1-4	0.023859812	0.008948138	down	-3.6370137	-1.86275
lnc-RELT-1	0.006345384	3.27E-04	down	-3.5700226	-1.83593

lnc-AC009365.3.1-1	0.02368607	0.008826657	down	-3.5443013	-1.8255
LINC01204	0.006072753	4.16E-05	down	-3.5343244	-1.82143
lnc-C3orf65-2	0.021547725	0.00758604	down	-3.4752212	-1.7971
TMEM92-AS1	0.0187362	0.005960597	down	-3.4705667	-1.79517
TMEM92-AS1	0.02413292	0.009130236	down	-3.4614983	-1.7914
lnc-OR51B6-1	0.006072753	1.99E-04	down	-3.432476	-1.77925
XLOC_l2_010831	0.011048292	0.002345771	up	175.93254	7.458879
LOC101929019	0.006072753	6.36E-05	up	157.90137	7.30288
lnc-PROC-2	0.006782571	5.96E-04	up	106.38419	6.73314
LINC00707	0.006072753	9.73E-05	up	102.69604	6.682237
lnc-KLF3-2	0.006782571	5.92E-04	up	85.95701	6.425543
LOC101928273	0.006782571	5.16E-04	up	75.716385	6.242534
lnc-MBIP-2	0.016182011	0.004592899	up	75.00083	6.228835
LINC00472	0.047808886	0.023497159	up	56.72619	5.825943
LINC00689	0.019741029	0.006390608	up	44.24582	5.467469
LINC01201	0.011613181	0.002558239	up	43.89887	5.456112
lnc-XRCC2-2	0.007946287	0.001069225	up	37.86802	5.242908
lnc-FRMD4B-2	0.010583176	0.002136054	up	35.8405	5.163519
lnc-KCNS3-4	0.032128397	0.01350263	up	35.603905	5.153964
LOC400743	0.006072753	2.69E-04	up	35.56976	5.152579
lnc-NOVA1-1	0.006417178	3.35E-04	up	32.76794	5.034213
LOC102724679	0.006782571	5.76E-04	up	25.94634	4.697459

lnc-TSHZ2-3	0.011666636	0.002660543	up	25.002253	4.643986
LINC00083	0.009106663	0.001384715	up	24.20005	4.596938
XLOC_l2_009136	0.021492854	0.007492922	up	22.522472	4.493293
XLOC_l2_015206	0.006072753	2.84E-04	up	21.463818	4.423835
LOC389033	0.014086007	0.003556886	up	20.588388	4.363759
lnc-SLC44A5-4	0.026331674	0.010318703	up	19.631796	4.29512
lnc-OLFM3-1	0.006072753	1.15E-04	up	18.451176	4.205641
LINC01405	0.006072753	1.59E-04	up	17.503967	4.12961
LOC101929610	0.008215214	0.001123508	up	15.966233	3.996952

Supplementary Table 3. Microarray results - RKO-CAPE 48h - The most differentially expressed coding genes (Top 25 most up/down regulated)

GeneSymbol	p (Corr)	p	Regulation	FC	Log FC
**OLAH**	0,001579187	2,46E-05	down	-48,92585	-5,612525
**TREM1**	0,004478552	0,001983222	down	-42,54717	-5,410991
**ROBO4**	0,00171886	2,59E-04	down	-29,56	-4,8855743
**MAP1LC3C**	0,001579187	7,63E-05	down	-22,148678	-4,4691486
**MFAP2**	0,00228211	6,42E-04	down	-17,965387	-4,167148
**CALCA**	0,001579187	7,02E-05	down	-16,922834	-4,0808992
**TSLP**	0,002065109	5,14E-04	down	-16,76373	-4,067271
**TREM1**	0,004216177	0,001813388	down	-14,381349	-3,846127
**AK9**	0,039669976	0,028219627	down	-11,569501	-3,5322547
**INSC**	0,001671252	2,25E-04	down	-10,8957815	-3,4456978
**SPX**	0,002276144	6,39E-04	down	-10,797617	-3,432641
**PRKACB**	0,008833086	0,004985571	down	-10,187157	-3,3486795
**KRTAP2-3**	0,001579187	3,46E-05	down	-9,676649	-3,2745075
**OLAH**	0,001579187	1,01E-04	down	-9,187096	-3,1996088
**C8orf46**	0,002749912	8,96E-04	down	-9,071577	-3,1813533
**NOS3**	0,002464793	7,40E-04	down	-8,98316	-3,167223

**MILR1**	0,01043405	0,006097918	down	-7,4712334	-2,9013464
**AOX1**	0,001579187	1,88E-05	down	-7,0945992	-2,8267212
**FLJ45513**	0,014579527	0,009117846	down	-6,67614	-2,7390141
**PRKACB**	0,015966631	0,010119983	down	-6,5985928	-2,7221584
**FRMD4A**	0,001579187	2,51E-05	down	-6,5961204	-2,7216177
**C1QTNF2**	0,002816881	9,51E-04	down	-6,522311	-2,7053833
**CDC20B**	0,03814312	0,027023628	down	-6,348848	-2,6664948
**CAPN15**	0,001579187	7,02E-05	down	-6,3338957	-2,663093
**XKR6**	0,001771149	3,05E-04	down	-6,3130727	-2,6583424
**GCNT3**	0,001579187	7,29E-06	up	61,9762	5,9536424
**AKR1B10**	0,002493334	7,52E-04	up	54,389973	5,765269
**AKR1B15**	0,001579187	1,26E-04	up	53,18661	5,732991
**AKR1B10**	0,001579187	6,36E-05	up	42,227005	5,400094
**TREM2**	0,001579187	4,29E-05	up	39,93845	5,3197064
**LCE1B**	0,001579187	1,39E-04	up	33,1162	5,049465
**SMOC2**	0,00223911	6,18E-04	up	32,821648	5,036576
**NEURL3**	0,001579187	6,16E-06	up	31,269915	4,9667034
**LCE1C**	0,001579187	1,28E-04	up	31,142782	4,960826
**SPANXA1**	0,001579187	1,31E-05	up	26,486244	4,7271714

**C3**	0,001579187	1,16E-04	up	23,706726	4,5672245
**CCDC30**	0,001579187	1,10E-05	up	21,461037	4,423648
**MCEMP1**	0,001579187	2,64E-05	up	21,457747	4,4234266
**IL7R**	0,001579187	6,14E-05	up	21,088312	4,3983717
**SLC2A9**	0,006832387	0,003581349	up	20,580654	4,363217
**PTPRR**	0,001693417	2,44E-04	up	19,944002	4,317883
**DCDC2C**	0,001579187	1,00E-04	up	19,243258	4,266281
**ENTHD1**	0,001579187	4,19E-06	up	18,826683	4,234707
**HECW2**	0,002213628	6,05E-04	up	18,776136	4,2308283
**ATP6V0D2**	0,001994183	4,66E-04	up	18,543621	4,212851
**AKR1B1**	0,001579187	5,16E-05	up	18,249523	4,189787
**ZNF365**	0,001579187	6,86E-05	up	18,176962	4,184039
**SPANXC**	0,001579187	3,66E-05	up	17,884104	4,160606
**SPINK1**	0,001579187	4,15E-05	up	16,886505	4,077799
**HSD3B1**	0,001579187	6,08E-05	up	16,552689	4,0489936

Supplementary Table 4. Microarray results - RKO- CAPE 48h - The most differentially expressed non-coding genes (Top 25 most up/down regulated)

GeneSymbol	p (Corr)	p	Regulation	FC	Log FC
**LINC01583**	0,001579187	1,22E-04	down	-81,64033	-6,35121
**lnc-RP11-597K23.2.1-2**	0,001579187	1,39E-04	down	-41,607433	-5,3787694
**lnc-TRAP1-1**	0,001579187	4,67E-05	down	-16,049162	-4,004426
**lnc-UMODL1-1**	0,002771783	9,14E-04	down	-12,169933	-3,6052494
**lnc-EXOC4-2**	0,001579187	2,19E-05	down	-11,532882	-3,527681
**LINC00571**	0,001817613	3,60E-04	down	-9,811036	-3,2944055
**lnc-MAGEE2-1**	0,001579187	2,10E-05	down	-8,139501	-3,0249403
**LINC01239**	0,007205072	0,003833325	down	-8,108405	-3,0194182
**WHAMMP3**	0,001579187	9,89E-06	down	-8,029205	-3,0052571
**lnc-RELT-1**	0,001669609	2,05E-04	down	-8,014538	-3,0026193
**LOC102724155**	0,00565877	0,002793672	down	-7,862545	-2,9749963
**LOC729291**	0,006432824	0,003299906	down	-7,84872	-2,9724574

**LPP-AS2**	0,001579187	1,20E-04	down	-7,677145	-2,9405699
**LOC101927851**	0,002065109	5,20E-04	down	-7,268925	-2,861742
**lnc-FARSB-1**	0,001867481	3,83E-04	down	-7,172267	-2,8424292
**LOC100505921**	0,003460268	0,001341475	down	-7,080228	-2,8237958
**LOC100506797**	0,003216045	0,001195582	down	-6,8833194	-2,7831044
**lnc-TOMM20-1**	0,003388387	0,001288217	down	-6,695801	-2,7432566
**lnc-C14orf118-1**	0,001910479	4,23E-04	down	-6,1958494	-2,631302
**lnc-GPN2-2**	0,001579187	1,33E-04	down	-6,1397657	-2,6181836
**SSR4P1**	0,020353222	0,013363872	down	-6,0038095	-2,5858781
**LOC100130938**	0,001579187	4,99E-05	down	-5,513946	-2,4630852
**LOC730961**	0,003840595	0,00158508	down	-5,5118127	-2,4625268
**LOC100128361**	0,003838185	0,001581343	down	-5,494001	-2,4578571
**UBAC2-AS1**	0,001579187	6,78E-05	down	-5,426688	-2,440072
**LOC344887**	0,001579187	4,76E-05	up	50,776936	5,6661015
**lnc-AC110619.2.1-1**	0,001579187	5,67E-05	up	39,038704	5,2868333
**NBEAP1**	0,001579187	3,37E-05	up	32,73886	5,0329323
**XLOC_l2_007178**	0,001669609	1,99E-04	up	30,64295	4,9374833
**lnc-RXRA-1**	0,001579187	3,58E-05	up	28,2205	4,8186717
**LINC01121**	0,001579187	5,29E-05	up	24,546476	4,617444

**XLOC_l2_004840**	0,001579187	5,69E-05	up	19,648142	4,296321
**XLOC_l2_015438**	0,001579187	1,21E-05	up	19,137852	4,258357
**LINC00520**	0,001579187	2,05E-05	up	18,394655	4,201215
**XLOC_l2_000384**	0,002621528	8,27E-04	up	17,267761	4,110009
**XLOC_l2_015438**	0,003447295	0,00132653	up	16,454601	4,040419
**lnc-SMARCAL1-3**	0,008063408	0,004439491	up	16,227282	4,0203495
**lnc-RP11-429E11.3.1-1**	0,001579187	3,17E-05	up	15,142077	3,9204912
**lnc-THNSL1-2**	0,001579187	4,59E-05	up	14,866286	3,8939724
**LINC00520**	0,001579187	3,34E-05	up	14,547396	3,862689
**lnc-ASMT-5**	0,002029816	4,94E-04	up	14,270984	3,835013
**lnc-ASMT-2**	0,001579187	9,51E-05	up	13,989347	3,8062568
**lnc-LRRC1-5**	0,001579187	2,00E-05	up	13,907864	3,797829
**MYH16**	0,011267884	0,00670142	up	13,793077	3,7858725
**LINC01021**	0,001579187	1,01E-04	up	13,599826	3,7655163
**LINC01336**	0,003938193	0,00164318	up	12,74844	3,6722488
**LOC101927501**	0,001579187	7,25E-05	up	11,763806	3,556283
**lnc-FAM54A-1**	0,001970166	4,54E-04	up	10,976446	3,4563391
**lnc-MMRN1-2**	0,004719967	0,002163833	up	10,551906	3,3994317
**LINC01021**	0,001579187	4,72E-05	up	9,993973	3,3210583

Supplementary Table 5. Microarray results – HCT-116- Kaempferol 48h - The most differentially expressed coding genes (Top 25 most up/down regulated)

GeneSymbol	p (Corr)	p	Regulation	FC	Log FC
**HIST2H2BE**	0,0473561	0,009852014	down	-9,692902	-3,2769287
**CNTN1**	0,02534135	1,11E-04	down	-6,111282	-2,611475
**ARHGEF10**	0,026350636	0,001500786	down	-5,638697	-2,4953618
**GREM1**	0,03374326	0,004088833	down	-5,4675674	-2,4508991
**RAET1E**	0,034570396	0,004389699	down	-5,4527025	-2,4469714
**ITGAX**	0,02534135	1,06E-04	down	-5,243344	-2,3904872
**TMCC2**	0,02534135	9,44E-04	down	-5,0222	-2,3283195
**STX1B**	0,027339293	0,002042017	down	-4,964615	-2,3116817
**PTGER1**	0,02534135	8,94E-04	down	-4,888847	-2,2894943
**IL6R**	0,02534135	7,00E-05	down	-4,8078237	-2,265384
**CHD2**	0,02534135	4,60E-04	down	-4,7667656	-2,2530107
**SCARNA14**	0,04319069	0,007907829	down	-4,7062597	-2,234581
**LCK**	0,038512416	0,005651039	down	-4,5386324	-2,1822577
**ELK1**	0,04638023	0,009274415	down	-4,406805	-2,139733
**ADAMTS2**	0,042844616	0,007687321	down	-4,359727	-2,1242378
**KRT32**	0,02534135	8,81E-04	down	-4,2178955	-2,0765233

**HYDIN**	0,02534135	3,74E-04	down	-4,1377115	-2,0488331
**PHTF2**	0,02534135	5,72E-04	down	-3,9463003	-1,9805007
**LRRC31**	0,02534135	5,99E-04	down	-3,9136195	-1,9685035
**RPS6KA2**	0,02534135	2,59E-04	down	-3,723631	-1,89671
**MTHFR**	0,02534135	0,001095554	down	-3,6464334	-1,866486
**HIST2H2AA4**	0,040738642	0,00670543	down	-3,5894442	-1,8437604
**ACVR1B**	0,043596003	0,008226963	down	-3,5222814	-1,8165102
**MC5R**	0,038976867	0,005970603	down	-3,4998796	-1,8073052
**SFXN5**	0,026531143	0,001722049	down	-3,471963	-1,7957516
**FAHD1**	0,02534135	6,85E-04	up	10,096209	3,3357418
**KHK**	0,02534135	6,26E-04	up	9,017088	3,1726615
**ADD2**	0,03446235	0,004254761	up	7,9913845	2,9984455
**KLHDC1**	0,031704348	0,003602006	up	7,113782	2,8306167
**SPATA31A3**	0,030224998	0,003225638	up	7,024714	2,8124394
**PATL2**	0,03578456	0,004810381	up	6,93076	2,7930136
**SKIDA1**	0,026844535	0,001884781	up	6,913162	2,7893457
**KLLN**	0,02534135	5,74E-04	up	6,4978914	2,6999717
**NCOA1**	0,031793337	0,003665247	up	6,3761406	2,6726835
**MTG1**	0,02826705	0,002488441	up	6,2979255	2,6548767
**ANKK1**	0,026350636	0,00164972	up	6,0723076	2,6022449
**MYB**	0,02534135	0,001177893	up	5,9620757	2,5758147
**SLC38A6**	0,038691305	0,005895361	up	5,9417367	2,5708847
**MUC20**	0,02534135	0,00105895	up	5,6228714	2,491307

**CTSW**	0,031704348	0,003601497	up	5,560731	2,4752746
**PPEF1**	0,042810224	0,007532851	up	5,443643	2,4445724
**ALB**	0,04319069	0,007925092	up	5,4255567	2,4397712
**CFAP44**	0,027993366	0,002360695	up	5,406207	2,4346168
**KCNH8**	0,02534135	9,67E-05	up	5,370005	2,4249234
**EDN1**	0,02534135	4,95E-04	up	5,2672567	2,3970518
**WDR78**	0,026844535	0,001855046	up	5,2577324	2,3944407
**PIGM**	0,02534135	7,47E-05	up	5,238993	2,3892896
**ZNF91**	0,043085422	0,007808268	up	5,1885514	2,3753319
**BCL2**	0,027699148	0,002242541	up	5,074681	2,343317
**ZNF107**	0,043242373	0,007971325	up	5,066422	2,3409672

Supplementary Table 6. Microarray results – HCT-116- Kaempferol 48h - The most differentially expressed non-coding genes (Top 25 most up/down regulated)

GeneSymbol	p (Corr)	p	Regulation	FC	Log FC
**lnc-KIF16B-1**	0,036473084	0,005009757	down	-12,701839	-3,6669655
**lnc-LRRC4C-4**	0,02534135	0,0010485	down	-11,648635	-3,542089
**FRMPD3-AS1**	0,02534135	8,90E-04	down	-10,439921	-3,384039
**lnc-SUSD1-2**	0,029763326	0,003025592	down	-8,530532	-3,0926356
**lnc-TMOD3-1**	0,028288204	0,002515368	down	-7,6161275	-2,9290576
**LOC100422737**	0,028990543	0,002730275	down	-6,5572433	-2,7130895
**lnc-AEBP2-2**	0,02756683	0,002205928	down	-6,2201014	-2,636938
**lnc-NFE2L2-1**	0,02534135	6,56E-05	down	-5,8385954	-2,5456214

**LOC100506860**	0,02534135	4,87E-04	down	-5,5801864	-2,4803133
**lnc-SMUG1-6**	0,026350636	0,001654536	down	-5,4269476	-2,440141
**LOC101928576**	0,027339293	0,002015736	down	-5,1418185	-2,3622787
**XLOC_l2_014827**	0,047236465	0,009688539	down	-4,7879767	-2,259416
**lnc-VGLL3-3**	0,02534135	0,001112861	down	-4,6565504	-2,2192616
**lnc-ARL6IP5-1**	0,026350636	0,001587701	down	-4,6174555	-2,207098
**NHS-AS1**	0,033065885	0,003930883	down	-4,4334636	-2,1484342
**MIR940**	0,038512416	0,005727714	down	-4,412425	-2,1415718
**lnc-TERF1-1**	0,036155473	0,004949406	down	-4,3317685	-2,1149561
**lnc-KIAA1919-1**	0,02534135	1,06E-04	down	-4,310634	-2,1079001
**lnc-ACTR6-1**	0,027991772	0,002308853	down	-4,010431	-2,0037572
**LOC101928295**	0,026195675	0,00133282	down	-4,005567	-2,0020065
**lnc-CETP-1**	0,02534135	1,66E-04	down	-3,9816654	-1,993372
**lnc-C6orf89-1**	0,04231574	0,007263483	down	-3,9261646	-1,9731207
**MIR181A1HG**	0,026350636	0,001675163	down	-3,8974864	-1,962544
**lnc-CETP-1**	0,04139119	0,006893679	down	-3,8822525	-1,9568939
**lnc-C17orf97-1**	0,02534135	1,64E-04	down	-3,8378112	-1,9402838
**LOC100130417**	0,02534135	5,93E-05	up	10,783989	3,430819
**LINC00476**	0,025656443	0,001222437	up	9,052451	3,1783085

**LINC01252**	0,02534135	3,00E-05	up	8,371915	3,0655577
**LOC255187**	0,042789795	0,007412583	up	7,665128	2,93831
**LOC100506797**	0,02534135	2,76E-04	up	7,2739396	2,862737
**XLOC_l2_000706**	0,043318804	0,008022492	up	6,8182955	2,769411
**lnc-QRSL1-1**	0,02534135	8,77E-04	up	6,7427826	2,753344
**lnc-C1orf180-1**	0,031793337	0,003630286	up	6,6681786	2,7372928
**LOC101927604**	0,02534135	1,71E-04	up	6,194266	2,6309333
**XLOC_l2_008221**	0,026270093	0,001459607	up	6,1303973	2,6159806
**lnc-CNN1-2**	0,029763326	0,003014101	up	6,089238	2,6062617
**C8orf37-AS1**	0,02534135	1,62E-04	up	6,0594893	2,5991962
**MGC39584**	0,036155473	0,004934802	up	5,9271703	2,5673435
**FLJ21408**	0,026195675	0,001357713	up	5,856481	2,550034
**LOC284561**	0,030017074	0,003143292	up	5,853198	2,549225
**CETN4P**	0,035389025	0,004730155	up	5,651288	2,4985797
**lnc-CRYGS-1**	0,04563223	0,009044587	up	5,5431533	2,470707
**LINC00704**	0,031793337	0,00365588	up	5,464017	2,449962
**METTL21EP**	0,0352537	0,00463832	up	5,4603367	2,4489899
**lnc-RALGDS-2**	0,026350636	0,001629264	up	5,44006	2,4436226
**LOC100996654**	0,029036827	0,002806144	up	5,189706	2,3756528

**LOC100507053**	0,030671496	0,003349806	up	5,089075	2,3474035
**lnc-WDR27-1**	0,04839221	0,010332068	up	5,0235276	2,3287008
**LOC101929566**	0,032026086	0,003707297	up	5,002261	2,3225803
**PAQR9-AS1**	0,03277476	0,003844665	up	4,9562745	2,309256

Supplementary Table 7. Microarray results – HCT-116- CAPE 48h - The most differentially expressed coding genes (Top 25 most up/down regulated)

GeneSymbol	p (Corr)	p	Regulation	FC	Log FC
CRYBA1	0,041469093	0,002424924	down	-4,676512	-2,2254329
CELF4	0,025024382	8,45E-05	down	-3,871132	-1,9527555
MIR146A	0,025024382	1,20E-04	down	-2,8968015	-1,5344608
SIM2	0,025024382	1,39E-04	down	-2,7410722	-1,4547403
SENP6	0,025024382	2,17E-04	down	-2,6717834	-1,417803
ZNF540	0,04196386	0,002562449	down	-2,4289608	-1,2803392
STS	0,03615443	0,001661115	down	-2,377853	-1,2496594
CDX4	0,03690205	0,001778587	down	-2,375038	-1,2479506
NCF1	0,043939844	0,003058813	down	-2,3330684	-1,2222285
KIF26B	0,025039753	4,85E-04	down	-2,2708411	-1,1832267
KRTAP11-1	0,032878034	0,001270262	down	-2,260814	-1,1768422
RTP4	0,02689703	6,11E-04	down	-2,2553782	-1,1733694
LCE2A	0,027882278	8,27E-04	down	-2,2428772	-1,1653507
KCNB1	0,04196386	0,002657949	down	-2,2100692	-1,1440915
SSPN	0,03886568	0,001961547	down	-2,1904836	-1,1312494
KCNH8	0,035935942	0,0015526	down	-2,0249922	-1,0179163
KRIT1	0,028212637	8,74E-04	down	-2,0235615	-1,0168967
FAM154A	0,04196386	0,002743959	down	-2,0138402	-1,0099492

**TSPAN32**	0,027882278	7,75E-04	up	8,239051	3,042478
**PCDH11X**	0,04196386	0,002586591	up	5,530422	2,4673896
**RBFOX1**	0,032376867	0,001185194	up	5,4777694	2,4535885
**PDXK**	0,027882278	8,39E-04	up	4,907237	2,294911
**TDGF1**	0,025024382	4,10E-05	up	4,438356	2,1500254
**CER1**	0,02689703	6,06E-04	up	4,3535604	2,1221957
**LRRC55**	0,027882278	6,88E-04	up	4,177579	2,0626671
**KALRN**	0,04469888	0,003169128	up	4,176717	2,0623693
**SPACA5**	0,025024382	2,63E-04	up	4,0076423	2,0027537
**COL20A1**	0,043175235	0,002963651	up	3,9427292	1,9791946
**SCN5A**	0,035634052	0,001518388	up	3,7770345	1,917254
**LOC391322**	0,041469093	0,002373308	up	3,6590219	1,871458
**OR5H6**	0,03690205	0,001782136	up	3,6346679	1,8618236
**SLC10A7**	0,039910782	0,002070655	up	3,3734539	1,7542264
**GPT**	0,045158517	0,00336481	up	3,3220308	1,7320654
**ITGAX**	0,027882278	7,88E-04	up	2,9871254	1,5787578
**NPR1**	0,025039753	5,01E-04	up	2,9327424	1,5522503
**DKK4**	0,025024382	4,01E-04	up	2,840456	1,5061226
**GRIP1**	0,047153924	0,003660051	up	2,6784513	1,421399
**NR1I3**	0,03690205	0,001765503	up	2,637651	1,3992536
**SLC26A10**	0,048030436	0,003809124	up	2,6119566	1,3851309
**SPDYE1**	0,025024382	2,19E-04	up	2,5971024	1,3769029
**HSF4**	0,025039753	4,36E-04	up	2,5785074	1,3665361

**TDRD1**	0,041469093	0,00233759	up	2,5728827	1,3633857
**SCX**	0,028677521	9,18E-04	up	2,532275	1,3404341

Supplementary Table 8. Microarray results – HCT-116- CAPE 48h - The most differentially expressed non-coding genes (Top 25 most up/down regulated)

GeneSymbol	p (Corr)	p	Regulation	FC	Log FC
lnc-KIF16B-1	0.03615443	0.001652193	down	-14.3639	-3.84437
lnc-LRRC4C-4	0.028904736	9.87E-04	down	-11.6881	-3.54697
FRMPD3-AS1	0.027882278	8.44E-04	down	-10.6187	-3.40853
lnc-AVEN-1	0.028904736	9.57E-04	down	-9.70665	-3.27897
LINC00310	0.025024382	3.32E-04	down	-8.58656	-3.10208
LOC100422737	0.04196386	0.002682922	down	-6.48938	-2.69808
XLOC_l2_001810	0.025024382	3.50E-05	down	-4.34525	-2.11944
lnc-KLF3-2	0.04196386	0.002688817	down	-3.54567	-1.82606
lnc-ZBED1-1	0.027882278	8.27E-04	down	-3.43828	-1.78169
C8orf37-AS1	0.039910782	0.002070902	down	-3.39386	-1.76293
lnc-CHADL-2	0.02750665	6.41E-04	down	-3.37283	-1.75396
lnc-PFKP-11	0.041469093	0.002370075	down	-3.19769	-1.67703
lnc-DDX20-2	0.029602015	0.001050959	down	-3.11532	-1.63938
XLOC_l2_013458	0.025791103	5.30E-04	down	-2.99443	-1.58228
lnc-MIER3-2	0.039910782	0.00208954	down	-2.97687	-1.5738
lnc-PALLD-2	0.043175235	0.002978768	down	-2.89273	-1.53243
lnc-ZNF382-1	0.029416146	0.001034121	down	-2.77981	-1.47499
lnc-IDS-1	0.025024382	3.17E-04	down	-2.70925	-1.4379

**lnc-JMJD7-PLA2G4B-2**	0.04196386	0.002788581	down	-2.69198	-1.42867
**lnc-PTPN3-2**	0.04196386	0.002560628	down	-2.39741	-1.26148
**lnc-KCNS3-4**	0.04196386	0.002787873	down	-2.32447	-1.2169
**lnc-RGL4-4**	0.027882278	7.04E-04	down	-2.31995	-1.2141
**lnc-C20orf96-3**	0.03690205	0.001785376	down	-2.28124	-1.18982
**lnc-HIATL1-5**	0.027882278	7.92E-04	down	-2.25934	-1.1759
**LOC101929608**	0.044761326	0.003237362	down	-2.15019	-1.10446
**MGC39584**	0.041469093	0.002313782	up	7.465664	2.900271
**lnc-MYO10-3**	0.04196386	0.002790369	up	7.085887	2.824948
**lnc-RASGRP3-5**	0.025024382	2.09E-04	up	6.352732	2.667377
**XLOC_l2_009887**	0.025024382	1.52E-04	up	6.26048	2.646273
**METTL21EP**	0.025024382	8.68E-05	up	4.646752	2.216223
**lnc-IVNS1ABP-2**	0.028306723	8.97E-04	up	4.423007	2.145027
**lnc-TRIML1-3**	0.025024382	6.45E-05	up	4.401312	2.137934
**DCTN1-AS1**	0.025024382	4.62E-05	up	4.390824	2.134492
**lnc-CAST-2**	0.041469093	0.002400197	up	4.296413	2.103133
**lnc-MYB-4**	0.032376867	0.001197375	up	4.274825	2.095865
**lnc-CNN1-2**	0.025024382	2.55E-04	up	3.995336	1.998317
**lnc-CCDC140-5**	0.048030436	0.003811674	up	3.976171	1.99138
**lnc-SDSL-1**	0.025024382	1.64E-04	up	3.685256	1.881765
**lnc-CX3CR1-2**	0.025024382	6.86E-05	up	3.631042	1.860384
**lnc-LSR-1**	0.039799664	0.002036391	up	3.500425	1.80753

**LOC102724193**	0.025024382	3.75E-04	up	3.401646	1.766233
**lnc-API5-4**	0.037654188	0.001861084	up	3.342033	1.740726
**XLOC_l2_008409**	0.025024382	2.53E-04	up	3.317344	1.730029
**LOC100506368**	0.025024382	1.13E-04	up	3.2161	1.685312
**LINC01252**	0.042335887	0.002871973	up	3.174506	1.666532
**XLOC_l2_000384**	0.025024382	9.76E-05	up	3.149525	1.655134
**LOC100507065**	0.044761326	0.003251269	up	3.0921	1.628587
**LOC101928058**	0.04196386	0.002804407	up	2.963253	1.567182
**lnc-LRRC58-1**	0.033768304	0.00137518	up	2.956415	1.563849
**C16orf86**	0.025024382	3.22E-04	up	2.940117	1.555873
